# Where do people purchase food? A novel approach to investigating food purchasing locations

**DOI:** 10.1186/s12942-017-0082-z

**Published:** 2017-03-07

**Authors:** Lukar E. Thornton, David A. Crawford, Karen E. Lamb, Kylie Ball

**Affiliations:** Institute for Physical Activity and Nutrition Research, School of Exercise and Nutrition Sciences, Deakin University, Geelong, VIC Australia

**Keywords:** Food environment, Food purchasing, Neighbourhood, Built environment, Geographic information system (GIS)

## Abstract

**Background:**

Studies exploring associations between food environments and food purchasing behaviours have been limited by the absence of data on where food purchases occur. Determining where food purchases occur relative to home and how these locations differ by individual, neighbourhood and trip characteristics is an important step to better understanding the association between food environments and food behaviours.

**Methods:**

Conducted in Melbourne, Australia, this study recruited participants within sixteen neighbourhoods that were selected based on their socioeconomic characteristics and proximity to supermarkets. The survey material contained a short questionnaire on individual and household characteristics and a food purchasing diary. Participants were asked to record details related to all food purchases made over a 2-week period including food store address. Fifty-six participants recorded a total of 952 food purchases of which 893 were considered valid for analysis. Households and food purchase locations were geocoded and the network distance between these calculated. Linear mixed models were used to determine associations between individual, neighbourhood, and trip characteristics and distance to each food purchase location from home. Additional analysis was conducted limiting the outcome to: (a) purchase made when home was the prior origin (n. 484); and (b) purchases made within supermarkets (n. 317).

**Results:**

Food purchases occurred a median distance of 3.6 km (IQR 1.8, 7.2) from participants’ homes. This distance was similar when home was reported as the origin (median 3.4 km; IQR 1.6, 6.4) whilst it was shorter for purchases made within supermarkets (median 2.8 km; IQR 1.6, 5.6). For all purchases, the reported food purchase location was further from home amongst the youngest age group (compared to the oldest age group), when workplace was the origin of the food purchase trip (compared to home), and on weekends (compared to weekdays). Differences were also observed by neighbourhood characteristics.

**Conclusions:**

This study has demonstrated that many food purchases occur outside what is traditionally considered the residential neighbourhood food environment. To better understand the role of food environments on food purchasing behaviours, further work is needed to develop more appropriate food environment exposure measures.

**Electronic supplementary material:**

The online version of this article (doi:10.1186/s12942-017-0082-z) contains supplementary material, which is available to authorized users.

## Background

The potential influence of neighbourhood factors on food purchasing and consumption has received growing attention, however empirical evidence remains inconclusive [1–3]. One of the reasons for this is that research has employed a range of different measures of food store access [4–6]. Two measures are commonly used: proximity to the nearest store, and the count of stores within a neighbourhood [1, 3, 7, 8]. Proximity measures typically ignore other store options nearby, whilst count measures are often limited to specific store types and apply a dichotomous categorisation to stores as being either accessible (within buffer) or not accessible (outside the buffer). Furthermore, when buffers are used there is little consensus on an appropriate buffer size, which is important as associations with food behaviours have been shown to be dependent on this [4].

Two additional limitations are common in many studies. First, exposure measures have been limited to a single context, most often within the residential neighbourhood. This ignores the multiple places people visit on a daily basis such as work, schools, and recreational settings. Second, existing measures also assume that all individuals within a particular neighbourhood have an equal ability to access facilities [9] and do not factor in other individual (e.g. cultural, socioeconomic, demographic and mobility) and environmental (e.g. public transport) factors which may influence food store choice [10]. As it stands, there are limited solutions to these problems as appropriate data on where people typically purchase foods to inform such measures are scarce.

A small but growing number of studies internationally have attempted to establish the spatial locations of habitual food purchasing patterns, both among adolescents [11, 12] and adults [13–19]. These studies have broadly concluded that many food purchasing behaviours occur beyond the boundaries of the residential neighbourhood or in stores that are not considered the most proximate to home. For example, Kerr et al. extracted food shopping trips from travel diary data in the US and found return trips between home, the food store, and home again were 5.37 mile (~8.64 km) in length and that trips to grocery stores were on average a distance of 4.67 mile (~7.52 km) from the trip origin, which may have been home, work, or some other location [19]. Whilst this body of work suggest that the access measures commonly applied may be too restrictive, further details related to food purchasing behaviours are required to help understand potential influences.

This paper presents findings from a novel data collection methodology which captured data on food purchasing locations and characteristics associated with food purchasing behaviours over a 2-week period. Data were mapped and distances calculated between the household address and food purchase locations. This study sought to explore purchase location relative to household address. Additional analysis examined whether purchase locations varied by characteristics of the individual, their neighbourhood and the food purchase trip. All food purchases, food purchases made when home was the trip origin, and supermarket purchases were examined separately. Those purchases made when home was the prior location may reflect habitual purchase behaviours that are less likely to be influenced by incidental travel (e.g. to social outings outside of their neighbourhood) and may be more likely to be influenced by neighbourhood food resources. Purchases made at supermarkets were also examined separately as supermarkets are the predominant location for food expenditure in Australia [20] and therefore have major influence on overall eating behaviours.

## Methods

### Study sites

This study was conducted within four local governments areas (LGAs) located to the east of the Melbourne CBD (Australia’s second largest city). Four Statistical Area Level 1 (SA1) administrative units were chosen within each LGA [average SA1 size within the four selected LGAs: 401 people (SD = 127), 0.215 km^2^ (SD = 0.35)]. The SA1s were sampled based on: (1) area-level socioeconomic disadvantage defined by the Australian Bureau of Statistics (ABS) Socio-Economic Indexes for Areas (SEIFA) Index of Relative Socio-Economic Disadvantage (IRSD) [two SA1s in the lowest quartile (low disadvantage termed “high socioeconomic status” (SES)) and two in the highest quartile (high disadvantage termed “low SES”)]; and (2) by access to supermarkets (high access: neighbourhoods with two or more Coles or Woolworths (two largest chains (~70% supermarket market share [21]) supermarkets within 2 km; and low access: neighbourhoods with no Coles/Woolworths supermarkets within 2 km). In each LGA, a SA1 was drawn from each quadrant of: low SES-low access; low SES-high access; high SES-low access; high SES-high access. This approach was employed to seek greater heterogeneity amongst participants in terms of socioeconomic and food environment characteristics. Whilst other supermarket chains (e.g. Aldi, IGA) and food store types (e.g. greengrocers) were present in the study region, the access measure was limited to the two dominant chains. However, even when limiting to these two chains, within one of the LGAs, no low SES-low access SA1s could be identified using the criteria above. In this instance the low SES SA1 located furthest (1.4 km) from the nearest (Coles/Woolworths) supermarket was used to represent low SES-low access in this LGA.

### Data collection

In October 2014, data collection material including a food purchasing diary and short survey was hand delivered to households within randomly selected streets in the sixteen selected SA1s (data collection tool available in Additional file 1). Supplementary targeted recruitment which involved additional survey deliveries occurred in quadrants of area-disadvantage/supermarket access until a minimum of ten valid food purchasing diaries in each quadrant were received. Fridge magnets were included in the package and were designed as a reminder to record food purchases. The delivered material was addressed to the main household food purchaser and this person was also required to complete a short questionnaire on their personal and household characteristics (e.g. age, sex, household composition, income). As a gesture of thanks, those who returned valid food purchasing diaries received a $20 gift voucher for a leading retailer and were entered into a prize draw for one of two $100 vouchers.

#### Food purchasing diary and survey

Within the food purchasing diary, participants were required to record details of all food purchases made over a 2-week period. This included foods made for immediate consumption, restaurant meals, and foods bought to be consumed later including packaged foods. Details to be reported included the date, name and address of store, where they were prior to making the purchase (home, work, other), primary mode of transport to the store (car, public transport, walk/cycle, other, or was home delivered), and what foods they purchased. The diary allowed for multiple purchases to be recorded on any given day and participants were to report if no food was purchased on a particular day.

The specific food items purchased could be recorded in one of two ways. First, participants could record what was purchased by ticking boxes against the categories listed in Additional file 2: Table S1. Second, participants had the option of attaching receipt data. Receipts were later coded against the same categories. Instructions noted that the purchase of multiple items from the same store should be recorded (e.g. hot fast food/takeaway and soft drink). Participants were asked to specify what the “other” item was when this box was checked. Many of these items were able to be recoded into one of the existing categories and therefore the “other” category was not examined further in analysis. Bottled water was also not examined due to the low number of purchases of this item.

### Sample and food purchase records

Fifty-six participants returned valid food purchasing diaries [quadrant break-down: low SES-low access (*n* = 11 participants); low SES-high access (*n* = 11); high SES-low access (*n* = 19); high SES-high access (*n* = 15)]. The majority of respondents were female (80%) with fewer participants in the youngest age bracket [18–34 years (20%); 35–54 years (36%); 55 years or over (41%)] (two participants did not report their sex or age).

The 56 participants recorded a total of 952 food purchases. The within-participant average total number of purchases made across the 2 weeks was 16.1 (SD = 7.6) at an average of 10.6 (SD = 5.2) different stores. Out of the 14 days, participants recorded purchases on an average of 9.0 (SD = 2.6) days. Whilst a slightly higher percentage of all purchases were recorded on Day 1 (11.8%) of the data collection period, purchases were generally spread evenly across the remaining days ranging from 5.3% of all purchases on Day 10 to 9.1% of purchases on Day 3. On Day 14, 6.9% of all purchases were recorded. This indicates that participants continued to report food purchases across the entire study period.

### Distance to food purchase location

Each participant’s household address (recorded in the consent form and stored separately to the survey) and where they made their food purchases were geocoded in ArcGIS 10.2 [22]. Store name and addresses recorded by participants were verified against online resources to supplement address information where required or to verify the full address. Of the 952 food purchases recorded, 916 were able to be geocoded (96.2%) with those not geocoded due to insufficient store details provided (*n* = 28) or because the purchase occurred interstate and was not considered a regular purchase location (*n* = 8). The shortest network path [8] between household address and food purchase location was calculated using the Network Analyst extension in ArcGIS. Pedestrian network paths were used for when the mode of travel was recorded as walking/cycling whilst street networks were used for all other modes.

### Statistical analysis

Data were examined for outliers and distances greater than 35 km (~21.7 mile) were excluded from analysis as these were considered locations that were less likely to be part of a regular routine (*n* = 24; 2.6% of geocoded purchases; distance range 47.3–248 km). This left a final sample of 893 food purchases. The distance between home and food purchase location was examined for all purchases and for two additional dependent variables: (1) distance between home and food purchase location for purchases made when home was reported as the prior location; and (2) distance between home and food purchase location for purchases within supermarkets. Supermarket purchases were defined as purchases within the four largest supermarket chains in Australia which have over 91% of the market share (Coles (market share 32.5%), Woolworths (37.3%), Aldi (12.1%), and IGA (9.7%) [21]). These stores were determined by the store name recorded by participants.

Descriptive statistics for the three different types of food purchase distances by individual and neighbourhood characteristics were generated along with a boxplot of distance from home by food item purchased. The descriptive statistics do not account for within-person clustering. A plot was also created of purchase distance from home for each purchase grouped by individual to visualise the distribution of distance from home.

To visualise the dispersion of purchase locations amongst individuals within the same neighbourhood (SA1), ArcGIS 10.2 was used to create a map with all purchase locations for a single SA1. Added to this were individual-specific standard deviation ellipses which represent the dispersion of purchase locations around the mean centre of these for each of the seven individuals who returned food purchasing diaries from this SA1. Standard deviation ellipses are a common way to represent dispersion of locations and are increasingly applied to studies exploring health behaviours or access to health services [14, 23, 24]. A one standard deviation ellipse was used which captures 68% of all food purchase locations for each individual. In the example SA1, the minimum number of unique purchase locations for an individual was five meaning a sufficient number of unique points were available to generate the ellipses. Household locations were not considered in the generation of these ellipses as the ellipses were created to visualise the dispersion of regular purchase locations which may or may not be near the household location. Food purchase locations are counted each time a purchase is made at that location. This essentially weights a location based on the frequency of trips to that location to purchase food.

Prior to inferential statistical analysis, all distance outcomes were log transformed to account for the skewness in the data and results are presented on these log transformed values. Linear mixed models were used to determine associations between individual, neighbourhoods, and trip characteristics and distance to each food purchase in Stata 14.0 [25] (Table 2). This three-level multilevel analysis examined each purchase accounting for the nesting of purchases within-individuals and within-areas (SA1s). Both the fixed effects and the level of clustering within-individuals and within-SA1s are reported. The clustering [intraclass correlation (ICC)] of purchase distance from home within-individuals and within-SA1s were estimated as part of the mixed effect models. The two ICC values presented are the proportion of the total variance in distance from home that is accounted for by the clustering within-individuals and within-SA1s. Essentially the ICC represents the correlation in the outcome within each cluster. One limitation when interpreting these is that the outcome assessed is distance from home and therefore it is not estimating if the same stores were visited but rather whether the stores visited were a similar distance from home. Two models were fitted for each of the three outcomes (Model 1: Null; Model 2: inclusive of individual characteristics (age, sex), neighbourhood characteristics (combined area-level disadvantage and supermarket access), and trip characteristics (location prior to purchasing (for all purchases and supermarket purchases only), mode of travel (for purchases made from home only), day of week). Mode of travel was only considered for purchases made when home was the prior location as this was a sensible trip origin to assess this variable. As the outcome assessed is distance from the home and not distance from the origin, results would have been biased if we included, for example, trips made from work during a lunch break where the mode of travel was walking but the actual purchase location is several kilometres from home. Both models were run on all non-missing values for each of the characteristics in Model 2 for comparability (all purchase *n* = 845; purchases made when home was the origin *n* = 460; purchases made within supermarkets *n* = 300). These two models allowed level of clustering within individuals and SA1s to be assessed prior to and after the addition of the individual, neighbourhood and trip characteristics.

## Results

### Descriptive results

**Fig. 1:**
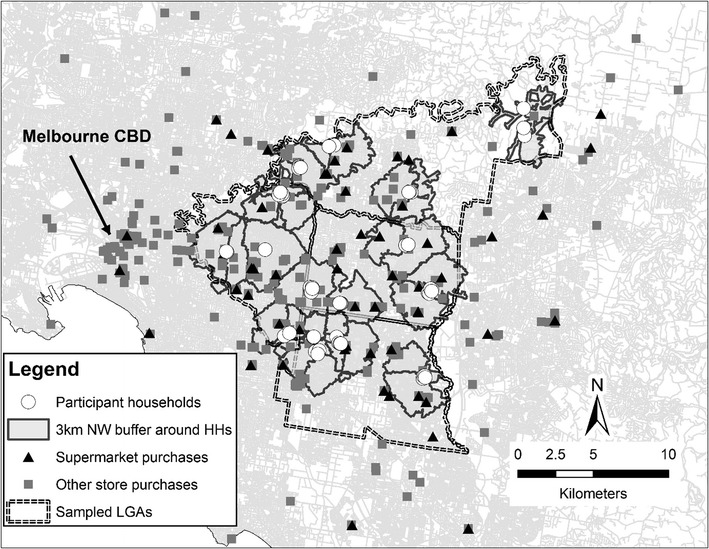
Location of participant households and food purchase locations

A total of 893 food purchases were considered in the descriptive analysis; 484 (54.2%) of these were made when home was reported as the prior location and 317 (35.5%) were made within supermarkets. Mapped household and food purchase locations are presented in Fig. 1.

Across all purchases, food purchases were found to take place a median distance of 3.6 km (IQR 1.8, 7.2) from participants’ homes, with the within-person median ranging from 0.3 to 16.8 km (Table 1). The median distance for purchases made when home was the prior location was only slightly lower than that for all purchases [3.4 km (IQR 1.6, 6.4)] whilst supermarket purchases were generally closer to home [2.8 km (IQR 1.6, 5.6)].

**Table 1:** Descriptive statistics for distance between home and food purchase locations by individual, neighbourhood, and trip characteristics

	All food purchases		Food purchases when origin of trip was home		Food purchases within supermarkets	
	n.	Median (IQR)	n.	Median (IQR)	n.	Median (IQR)
Distance from home (km) (if ≤ 35 km)	893	3.64 (1.82, 7.19)	484	3.40 (1.60, 6.37)	317	2.79 (1.61, 5.59)
Within-person median distance from home (range)	56	min: 0.35 max: 16.81	55	min: 0.33 max: 22.96	56	min: 0.29 max: 15.33

	n.	% of purchases	n.	% of purchases	n.	% of purchases
*Distance from home categories (km)*						
≤1	93	10.1	60	12.3	54	16.5
>1–2	160	17.5	97	19.9	57	17.4
>2–3	93	10.1	54	11.1	49	15.0
>3–5	196	21.4	100	20.5	67	20.5
>5–10	184	20.1	113	23.2	77	23.5
>10–20	151	16.5	51	10.5	13	4.0
>20–35	16	1.8	9	1.9	0	0
>35 (excluded from analysis)	23	2.5	3	0.6	10	3.1

	n.	Median distance from home (IQR)	n.	Median distance from home (IQR)	n.	Median distance from home (IQR)
*Age* ^*a*^						
18–34 years	173	4.35 (1.61, 14.35)	85	3.71 (1.48, 7.82)	44	2.16 (0.33, 4.03)
35–54 years	373	3.54 (1.70, 5.98)	164	2.67 (1.61, 5.67)	118	2.62 (1.70, 5.27)
≥55 years	318	3.49 (1.93, 6.74)	219	3.53 (1.96, 6.74)	146	3.38 (1.85, 5.59)
Missing	29	6.61 (1.32, 9.60)	16	6.58 (1.30, 12.57)	9	6.61 (1.27, 9.09)
*Sex* ^*b*^						
Female	743	3.54 (1.87, 6.56)	401	3.44 (1.70, 5.98)	261	3.29 (1.82, 5.60)
Male	121	5.09 (1.70, 12.43)	67	2.28 (0.63, 8.66)	47	1.70 (0.63, 2.18)
Missing	29	6.61 (1.32, 9.60)	16	6.58 (1.30, 12.57)	9	6.61 (1.27, 9.09)
*Neighbourhood characteristics*						
Low SES-Low access	200	2.78 (1.31, 4.71)	82	2.09 (1.29, 3.74)	50	2.08 (1.22, 2.85)
Low SES-High access	181	4.03 (0.87, 12.43)	92	2.63 (0.71, 4.35)	58	0.71 (0.63, 2.79)
High SES-Low access	309	5.60 (3.40, 8.43)	192	5.59 (3.38, 7.19)	122	5.59 (3.47, 6.56)
High SES-High access	203	3.17 (1.82, 5.22)	118	2.45 (1.65, 5.03)	87	2.23 (1.56, 3.29)
*Origin prior to making purchase*						
Home	484	3.40 (1.60, 6.37)	–	–	202	2.62 (1.56, 5.59)
Work	164	5.13 (3.13, 14.83)	–	–	33	2.85 (1.52, 5.13)
Other	226	3.62 (1.96, 7.38)	–	–	74	3.38 (1.90, 4.55)
Missing	19	2.64 (0.85, 6.56)	–		8	2.36 (0.67, 6.12)
*Travel mode when origin was home* ^*c*^						
Car	–	–	386	3.74 (2.08, 6.65)	–	–
Public transport	–	–	16	4.35 (4.35, 4.62)	–	–
Walk/cycle	–	–	73	0.74 (0.63, 1.58)	–	–
Missing			7	2.09 (1.82, 11.41)		
*Day of week*						
Weekday	629	3.54 (1.82, 6.74)	307	3.37 (1.58, 5.88)	223	2.62 (1.47, 5.13)
Weekend	264	4.33 (1.82, 8.59)	177	3.74 (1.65, 8.66)	94	3.46 (1.82, 5.75)

^a^Number of participants by age group: 18–34 years *n* = 11 (19.6%); 35–54 years *n* = 20 (35.7%); ≥ 55 years *n* = 23 (41.1%); missing *n* = 2 (3.6%)

^b^Number of participants by sex: female *n* = 45 (80.3%); male *n* = 9 (16.1%); missing *n* = 2 (3.6%)

^c^Results not shown for response categories where fewer than 10 purchases by travel mode (other *n* = 0; home delivery *n* = 2 [4.56 (IQR 3.61, 5.51)]

**Fig. 2:**
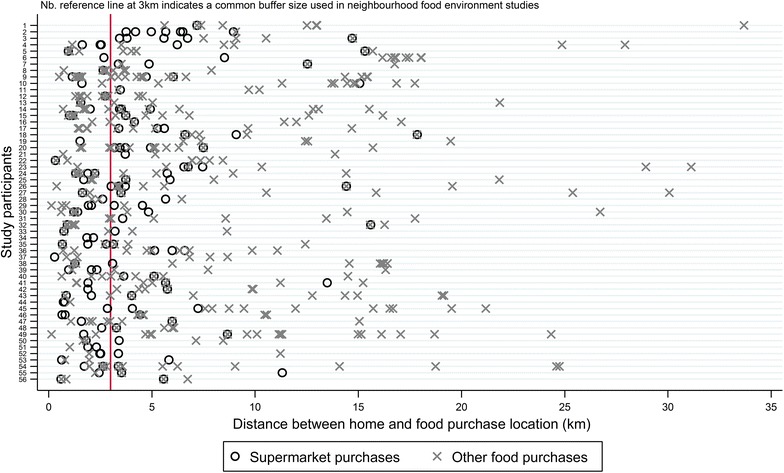
Distance from home of all food purchase locations for each participant

Over 60% of all food purchases occurred beyond 3 km of participant’s homes (Table 1). This is demonstrated in Fig. 2 with the 3 km distance (a commonly used buffer size in studies of food environment exposure) marked on this graph to highlight the food purchases taking place beyond this distance. Two participants made all purchases during the 2 weeks within 3 km of their home, whilst six participants made all of their purchases more than 3 km from their home.

**Fig. 3:**
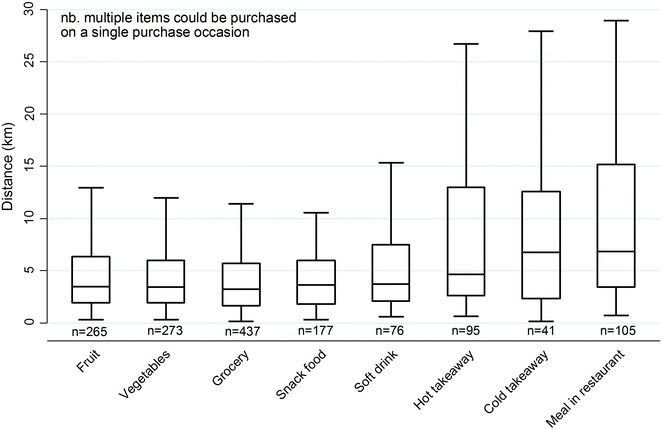
*Boxplot* of distance from home to food purchase location by item purchased

Differences in distance between home and purchase location by individual, neighbourhood and trip characteristics are also detailed in Table 1 and are further explored in the multilevel analysis accounting for within-person and within-neighbourhood clustering. Variation in distance to food purchase location from home was also observed by different food items purchased (Fig. 3). The median distance between home and food purchase location was shortest for grocery items (3.2 km; IQR 1.6, 5.7); however, this distance was similar for other fresh and packaged food items (fruit, vegetables, snack food, and soft drink) which reflects the fact many of these items were purchased concurrently in supermarkets. Median distances were greater when the item purchased was hot takeaway (4.6 km; IQR 2.6, 13.0), cold takeaway (6.7 km; IQR 2.3, 12.6), and meals in restaurants (6.8 km; IQR 3.4, 15.2).

**Fig. 4:**
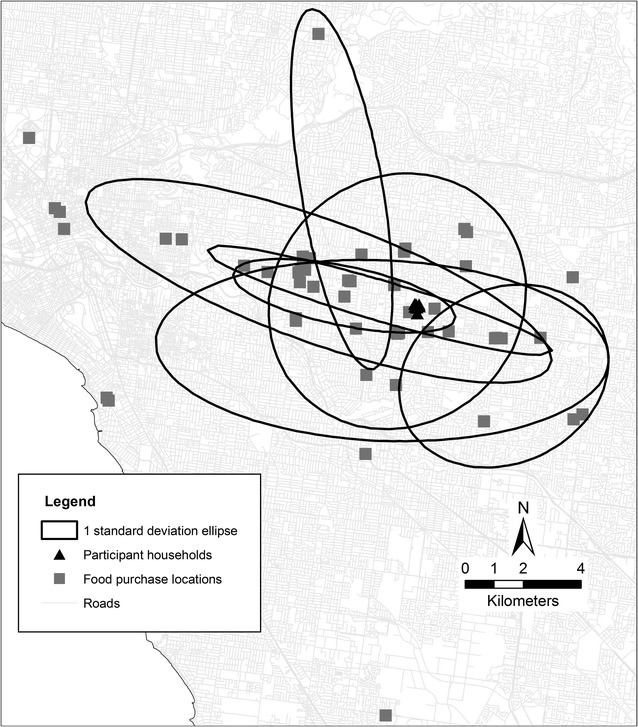
Food purchase locations and a one standard deviation ellipse around the mean centre of purchase locations for seven individuals in a single sampled neighbourhood (SA1)

Figure 4 presents the food purchase locations for all seven individuals living within a single high SES-low access SA1. The standard deviation ellipses presented in this figure highlight the dispersion of purchases locations within individuals but also the similarities and differences in regular purchase locations between individuals who live within close proximity of each other.

### Multilevel analysis

For all purchases and for purchases made when home was the prior location, there was evidence to suggest that the distance between home and the food purchase location was greater amongst the youngest age group compared to those aged 55 years and over (Table 2). For the purchases made at supermarkets, age was not associated with distance from home, however supermarket purchases made by men were closer to home than supermarket purchases by women.

**Table 2:** Linear mixed models for distance from home to food purchase location (log transformed)

All purchases		Purchases made when home was the origin		Purchases made at supermarkets	
Model 1: Null (*n* = 845)		Model 1: Null (*n* = 460)		Model 1: Null (*n* = 300)	
Intraclass correlation (%)		Intraclass correlation (%)		Intraclass correlation (%)	
Individual	18.4	Individual	13.3	Individual	7.4
SA1	20.6	SA1	28.9	SA1	60.6

	Model 2: All characteristics (*n* = 845)		Model 2: All characteristics (*n* = 460)		Model 2: All characteristics (*n* = 300)	
	Coef. (95% CI)	p. value	Coef. (95% CI)	p. value	Coef. (95% CI)	p. value
*Age (years)*						
18–34	0.56 (0.13, 0.99)	0.011	0.40 (0.02, 0.79)	0.041	0.14 (−0.21, 0.49)	0.437
35–54	0.21 (−0.12, 0.54)	0.219	−0.16 (−0.45, 0.14)	0.292	0.01 (−0.25, 0.27)	0.938
55+	Ref.		Ref.		Ref.	
*Sex*						
Female	Ref.		Ref.		Ref.	
Male	0.10 (−0.25, 0.45)	0.572	−0.06 (−0.37, 0.24)	0.684	−0.34 (−0.60, −0.08)	0.010
*Neighbourhood*						
Low SES-Low access	Ref.		Ref.		Ref.	
Low SES-High access	−0.25 (−0.94, 0.44)	0.477	−0.26 (−0.85, 0.32)	0.376	−0.46 (−1.33, 0.40)	0.293
High SES-Low access	0.70 (0.04, 1.36)	0.037	0.57 (0.07, 1.08)	0.025	0.75 (−0.08, 1.58)	0.077
High SES-High access	−0.09 (−0.76, 0.59)	0.800	−0.19 (−0.72, 0.34)	0.477	−0.13 (−0.96, 0.71)	0.767
*Location prior to food purchase*						
Home	Ref.		n/a		Ref.	
Work	0.59 (0.42, 0.76)	<0.001	–		0.36 (0.15, 0.58)	0.001
Other	0.13 (−0.01, 0.27)	0.075	–		0.12 (−0.03, 0.27)	0.124
*Mode of travel from home*						
Car	n/a		Ref.		n/a	
Public transport	–		0.18 (−0.29, 0.65)	0.445	–	
Walk/cycle	–		−1.15 (−1.38, −0.92)	<0.001	–	
*Day*						
Weekday	Ref.		Ref.		Ref.	
Weekend	0.17 (0.05, 0.30)	0.008	0.14 (−0.01, 0.29)	0.06	0.10 (−0.04, 0.27)	0.153

Intraclass correlation (%)		Intraclass correlation (%)		Intraclass correlation (%)	
Individual	14.5	Individual	9.5	Individual	6.1
SA1	16.8	SA1	11.6	SA1	52.8

Nb. n. for both Model 1 and Model 2 based on non-missing values in Model 2 for comparability

Compared to those in low SES-low access SA1s, purchases made by those in high SES-low access SA1s were a further distance from home for all purchases and purchases made when home was the prior location. Purchases were further from home for all three outcomes for those in high SES-low access SA1s compared to low SES-high access SA1s (Additional file 2: Table S2). Conversely, amongst SA1s deemed high SES-high access, purchases were nearer to the home when compared to purchases made by those in high SES-low access SA1s for all outcomes. Amongst those in low SES SA1s, there was no difference in purchase distance from home between those in high access compared to low access neighbourhoods.

When the workplace was the prior location compared to when home was the prior location, all purchases and supermarket purchases were further from home. For purchases made when home was the origin, mode of travel was examined with trips made by walking found to be significantly shorter than trips made by car. For all purchases and purchases made when home was the prior location, purchases made on the weekend were further from the home compared to purchases on the weekday. No difference in weekend compared to weekday was found for supermarket purchases.

### Intraclass correlations

The within-person and within-neighbourhood (SA1) correlations were assessed for both models across the three outcomes. For all purchases in Model 1, the ICC for individuals (18.4%) and for SA1s (20.6%) were similar. The inclusion of individual, neighbourhood, and trip characteristics in Model 2 accounted for some of this ICC with individual ICC reducing to 14.5% and SA1 ICC to 16.8%. For purchases made from home and supermarket purchases, the amount of clustering was higher within SA1s than within individual in the null models. For purchase made from home, the individual and SA1 ICC were more similar when accounting for individual, neighbourhood and trip characteristics (individual ICC: 9.5%, SA1 ICC: 11.6%). For purchases made in supermarkets, the SA1 ICC reduced from 60.6% in Model 1 to 52.8% in Model 2 but still suggested a much higher degree of clustering than within-individuals (6.1%).

## Discussion

The study builds upon a developing evidence base that demonstrates that the neighbourhood food environment, as traditionally defined, is inadequate for capturing important locations where individuals are exposed to and purchase food. Further, it has shown that distance from home to purchase location varies by the type of food being purchased and also by individual, neighbourhood and trip characteristics. This study’s results are supported by prior work from the US. Kerr et al. report the average travel distance (from any origin) to a grocery store to be 4.67 mile (~7.52 km) [19]. They also reported that trips made from home, to a food store, and back home again were an average distance of 5.37 mile [19], or a one way trip of approximately 2.69 mile (~4.32 km). In this present study median distance is reported rather than average due to skewed distribution of the data. The median distance found in the study was 3.40 km but the average distance was 5.03 km.

The sampled neighbourhoods were a mix of those defined as having access to the two major chain supermarkets (Coles and Woolworths) nearby and those without. However, when considering all four major chains (Coles, Woolworth, Aldi and IGA) only three of the 56 participants lived further than 3 km of any supermarket and it is plausible that the presence of a supermarket may be a proxy for the presence of other food retailers. It is therefore unlikely that the lack of nearby food retailers was the key reason that over 60% of all food purchase and over 50% of supermarket purchases occurred more than 3 km from the home.

Distances from home were greatest when the food being purchased was hot takeaway food, cold takeaway food, or meals within sit down restaurants. The location of both the workplace and social activities are likely to be key contributors to this as would an individual’s preference for a particular cuisine which may require them to travel a greater distance. US studies have also reported a higher distance to sit down restaurants compared to other store types [17, 19].

In the present study greater distance to food purchase locations was observed among younger age groups which perhaps indicates higher levels of daily mobility. Compared to trips made when home was the origin, distance between home and the purchase location was unsurprisingly greater when workplace was reported as the origin. It is likely this was largely dictated by workplace location. Whilst prior work by this study’s authors did not find evidence that the relationship between food stores near home and eating behaviours differed by work status [26], Zenk et al. [14] have previously shown that those employed have larger activity spaces than those not in the labour force suggesting that use of stores further away is more likely. Although that study took place in the US, the clustering of employment opportunities outside of suburban residential areas across Melbourne means this is also likely to be the case in this sample.

Purchases on the weekend were also a greater distance from home than purchases on weekdays (though not to the same extent as the origin of trip differential). Non-work day purchases were also a greater distance from home in Kerr et al.’s study [19]. Purchase locations on the weekend, where more free time is expected, may be more heavily influenced by store preferences and the location of social outings whereas weekday purchase may be determined by time scarcity and convenience.

When home was the prior location, food purchase locations reached by walking or cycling were a median distance of 3 km closer to home than purchase made using a car. This indicates that those engaging in active forms of transport more often used local food stores than those who travelled by car. However, it is not possible to determine whether specific purchases made by active travel were due to personal preference or because of lack of access to a motor vehicle at the time of purchase. Whilst the benefits of active transport are well established, if purchase location was restricted because of limited vehicle access then this has the potential to result in less healthy food purchases [27].

The linear mixed model results show the neighbourhood of residence (combined area SES and supermarket access) was associated with food purchase distance from home. Participants from high SES-low access SA1s purchased food further from home than participants from each of the other three sampling quadrants. This indicates that whilst nearby supermarkets (and potentially other food stores) may have been located further away, the high SES status of these neighbourhoods could mean that higher levels of employment or greater personal means (e.g. access to a motor vehicle) facilitated the ability and willingness to travel further for food purchases.

Differences in food purchase locations presented in Figs. 3 and 4 highlight that the utilisation of standard food environment exposure measures within a set boundary from household locations may not result in the generation of new and important advances in the field. Whilst the purchase locations and standard deviation ellipses presented in Fig. 4 indicate the home is an important ‘anchor point’ [9, 28, 29] around which purchases take place, individual variations were apparent. Given differences in individual characteristics, it should not be expected that residents utilise their neighbourhood in the same way. Further, a number of often unmeasured environment differences would also impact on the use of neighbourhoods for food purchasing purposes. For example, neighbourhoods with two supermarkets may differ with regards to a number of other important environmental characteristics (e.g. crime, public transport, walkability) meaning individual use of local supermarkets between these neighbourhoods would likely differ. For this reason, there needs to be a greater emphasis on both individual- and environmental-level moderators.

Kwan has previously described the need to give further consideration to individuals when considering contextual effects [30, 31]. Sharkey and Faber have previously called on researchers investigating residential contextual effects to be more flexible in their approach and answer the questions of where, when, why, and for whom do residential contexts matter [32]. There is an increasing body of food environment research adapting such an approach to investigate where and, in some cases, when food environments matter [12, 33–38]. However, future research needs to continue to evolve to ensure the equally important questions of why and for whom are also answered.

### Strengths

The novel data collection method used highlights the potential opportunities provided by studies that collect data on behaviour location. This study was strengthened by the collection of food purchasing data over a 2-week period which allowed for the capture of regular and occasional purchase behaviours. Food purchasing data provides an insight into how individuals interact with the environment and removes assumptions associated with studies that link neighbourhood exposure measures to consumption or health outcomes. Whilst it is not possible to verify missing purchases, the fact participants continued recording data across all fourteen days and that multiple purchases on each day were often recorded indicates good compliance. There were very few problems with the food store data provided (name/address) meaning that over 96% of purchase locations were able to be identified.

### Limitations and considerations for future research

This study was limited to a single region of Melbourne, Australia. Whilst an attempt was made to diversify the sample through choosing sample locations that differed by levels of socioeconomic disadvantage and access to major supermarket chains, future work would benefit from being undertaken across a more expansive and diverse area with regards to both population characteristics and the local environments.

A larger study involving more participants would allow for a deeper investigation into the role of individual- and household-level modifiers. Individual and household factors such as age, motor vehicle ownership, disability, family composition (e.g. presence of young children in the household), hours worked per week, workplace location and food preferences are all likely to influence which food stores are visited. Greater consideration of these and other environmental factors (e.g. walkability of neighbourhoods, provision of public transport, and in-store factors such as product range, quality and price) would allow us to understand why two people living in the same neighbourhood access different food stores.

This study objective was to capture the food purchasing locations of the main household food purchaser and consequently the study material was addressed to this person. Whilst this approach potentially captured a large portion of food purchased for consumption by other household members, independent purchases by other household members were not recorded. The completion of the diary by all members of the household would allow for both individual and household purchasing patterns to be assessed.

Future studies should also consider collecting further (precise) address information of the origin of trips (e.g. workplaces). Whilst the addition of GPS data would help to capture this information, the simple reporting of additional address information on other key origins would provide more context into why purchases are occurring where they do. Whilst this study collected data on work postcode, the large size of these areal units did not provide a meaningful location to be able to calculate purchase distance between work and purchase location when work was the origin. Prior studies suggest food stores outside of the residential context for example, near workplaces, may be important to food behaviours [26, 39]. Therefore it is important that precise address data on workplaces and other frequently visited locations are collected in future studies.

The food categories collected could be further refined (e.g. any vegetables instead of fresh vegetables only which excluded frozen options). This would potentially allow a more detailed analysis on the impact of the food purchase location on the healthiness of food purchases.

Finally, it is acknowledged that the definition of supermarket was different for access (Coles and Woolworths) and purchases (Coles, Woolworths, Aldi and IGA). However, our access measure was based on the two most dominant chains which have ~70% market share and it is unlikely a sufficient neighbourhoods that did not have supermarket access would have been identified if additional supermarket chains were included. Whilst the dominant supermarket chains were used as a proxy for food store access in this study, it is by no means a perfect measure. Future work should pay particular attention to the development of access measures prior to sampling to ensure even greater heterogeneity in neighbourhood food environment measure. This will require access to detailed food retail datasets with accurate and complete data and a range of food store categorisations. Other environmental heterogeneity could also be introduced through the inclusion of other metrics such as walkability.

## Conclusions

Through the collection of food purchasing locations this study has been able to demonstrate that many food purchases occur beyond what is commonly defined as the residential neighbourhood food environment. Further, results highlight the potential role of individual and neighbourhood characteristics as an influence on food purchase locations. This study’s methods and results can inform our thinking on the appropriateness of using narrowly-focussed neighbourhood exposure measures when trying to understand the associations between food environments and food purchasing behaviours.

## Additional file

**Additional file 1.** Data collection tool.

**Additional file 2.** Supplementary tables.
