# Supplementary material for: Where do people purchase food? A novel approach to investigating food purchasing locations
Source: Int J Health Geogr. 2017 Mar 7;16:9. doi: 10.1186/s12942-017-0082-z (PMC5341394; doi:10.1186/s12942-017-0082-z)

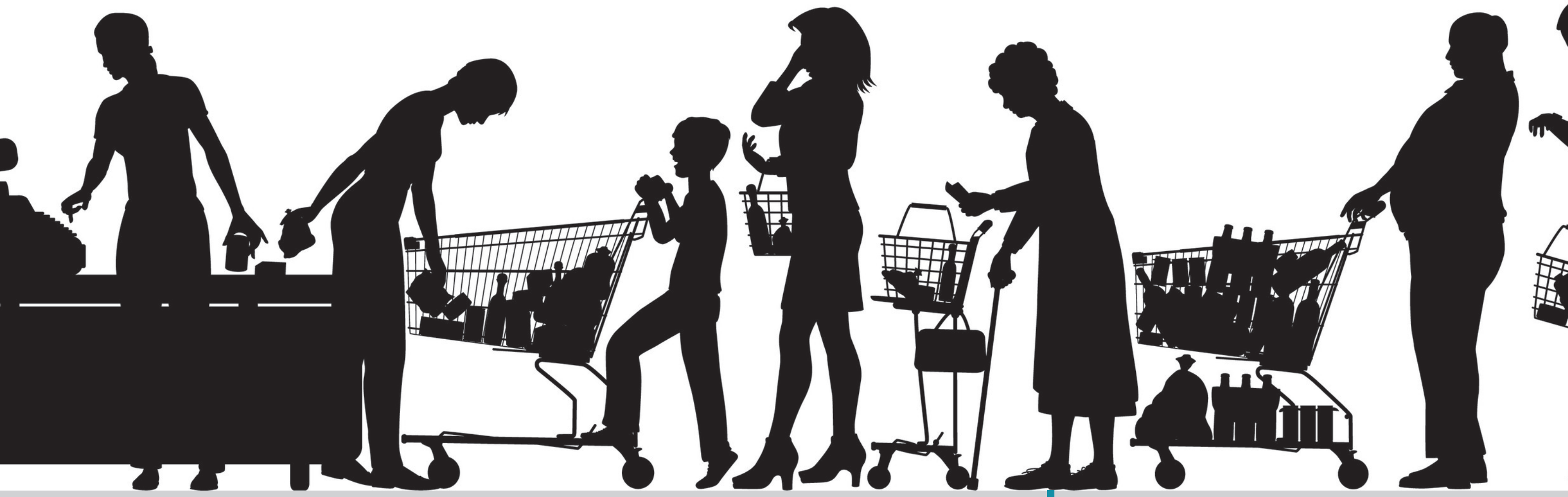

2014

C-PAN

Centre for Physical Activity and Nutrition Research

FOOD PURCHASING STUDY

CRICOS Provider Code: 00113B

DEAKIN UNIVERSITY AUSTRALIA

Please Read

This booklet contains a number of important sections:

- **Food Purchasing Diary instruction manual** – This contains detailed instructions on how to fill in the food purchasing diary.

- **Food Purchasing Diary –** This is what you will fill in each day for the duration of the study (14 consecutive days).

- **Individual Questionnaire –** Please answer these questions about yourself and your household in the space provided.

Once you have completed the food purchasing diary and the questionnaire please send them back to us in the reply-paid envelope (provided). Please note that you **DO NOT** need a stamp for this.

If you have agreed to also be involved in the GPS component of the study please wait for the device to be delivered prior to beginning the food purchasing diary. The diary and the device can be returned together.

For any questions or concerns regarding the study please do not hesitate to contact us at:

Dr Lukar Thornton
School of Exercise and Nutrition Sciences
Deakin University
221 Burwood Highway, Burwood VIC 3125
Phone: 03 9244 5029
Email: food_purchasing@deakin.edu.au

Food purchasing diary: Instruction manual

Dear participant,

Thank you for agreeing to be involved in this important study. Below are the instructions on filling in the food purchasing diary.

If you have any questions please email: **food_purchasing@deakin.edu.au**

How to fill in the food purchasing diary:

A separate sheet is included for each day with four rows available to record four separate food purchases for each day. If you make more than four purchases on any day, please record the additional purchases on the additional purchases sheet provided after Day 14. You can start the diary on any day of the week and please remember to fill it in for 14 consecutive days.

On each day please record food purchases made at different stores as a separate food purchase (e.g. if on one trip you visited the supermarket and the bakery, record these as two separate food purchases).

Two separate visits to the same store on the same day (e.g. a café in the morning for breakfast and again for a meal in the evening) are also considered separate food purchases.

If no food purchases are made on a particular day, please tick the box at the top of that day indicating this.

Receipt

To begin with, please indicate whether you obtained a receipt for the food purchase. If you are attaching the food purchase receipt, please note on the receipt the date and food purchase number it relates to (see below).

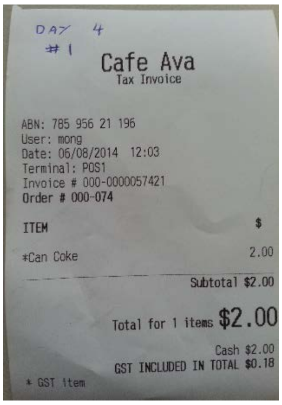

What was the name of the store where the food was purchased and the address (store name, street number, street name or location, and suburb):
In this section please enter as much information about the location where you purchased food as you can. The minimum information of store name, street and suburb are required.

Where were you prior to making this food purchase?
In this section please tell us where you were before you travelled to make this food purchase. That is, were you at home, at work, or were you at some other location?

Primary mode of travel from the prior location to the food store:
When you left the prior location you recorded to visit the food store, did you primarily travel by car, public transport, walk or cycle, other means such as a taxi, or was the food home delivered? If groceries items are purchased but to be home delivered at a later date, please still record the items on the day of purchase.

What food was purchased?:
Ten categories of food items are provided for you to select from. Examples of foods that fall within each category are as follows:

Food category	***Examples of food items***
**Fresh fruit**	All fresh fruit items that are either as individual items (e.g. a single apple) or as bulk (e.g. 6 pack of Kiwi fruit). Do not included canned or dried fruit in this category.
**Fresh vegetables**	All fresh vegetable items that are either as individual items (e.g. a lettuce) or as bulk (e.g. 1kg of carrots). Do not included canned or dried vegetables in this category.
**Grocery items**	This includes household grocery items such as bread, milk, cheese, pasta, rice, tinned fish, butter, canned fruit, meat, breakfast cereal, yoghurt etc.
**Snack food**	Potato/corn chips, chocolate, confectionery, muesli bars, popcorn, pretzels, pastries (e.g. cinnamon bun), ice cream etc.
**Soft drink**	This includes all soft drinks (both sugar sweetened and diet varieties), sports drinks, energy drinks etc.
**Bottled water**	This includes all bottled natural water (still, free of flavours etc).

**Hot fast food/takeaway**	Examples include hot chips, pizza, meat pie, hamburger, souvlaki, fish & chips, fried/roast chicken, fried rice, curry, etc.
**Cold takeaway**	Examples include sandwiches, salads, sushi etc.
**Meal within sit down restaurant**	This includes all eat in occasions in a full-table service sit down restaurant. It does not include occasions such as eating within a McDonalds restaurant (which should be recorded under hot fast food/takeaway)
**Other**	Please specify any other item that is not in the listed above here. Additionally, if you are unsure how to classify an item, please list it here.

The purchase of multiple items from the same store should be recorded (e.g. Hot fast food/takeaway and soft drink).

Amount Spent:
In this section please indicate the total amount spent for each food purchase number. Where possible please only note the amount spent on food and drink items.

See the example below on how to fill out the food diary.

Day 1: 30/7/2014

No food purchase made on this day: ☐

**Food purchase (FP) number/ receipt attached**<br>*(report for each different store visited)*	**Store name/address**<br>(<u>minimum information:</u> store name, street/location, suburb)	**Where were you prior to making this food purchase?**	**Primary mode of travel from the prior location to the food store.**	**What food was purchased**<br>Please keep and return receipt and note which day and food purchase number it relates to<br>**OR**<br>Fill in checklist below	**Amount spent $**
**# 1**<br>Receipt attached:<br>☐ Yes<br>☑ No	Store name: Café Ava<br>Street number:<br>Street name/location: Deakin University<br>Suburb: Burwood	☐ Home<br>☑ Work<br>☐ Other (please specify) ____________.	☐ Car<br>☐ Public transport<br>☑ Walk/cycle<br>☐ Other<br>☐ Home delivery	☐ Fresh fruit<br>☐ Fresh vegetables<br>☐ Grocery items (e.g. bread, milk, pasta)<br>☐ Snack food (e.g. chips, chocolate)<br>☐ Soft drink<br>☑ Bottled water<br>☐ Hot fast food/takeaway (e.g. KFC, Chinese, pizza)<br>☑ Cold takeaway (e.g. sandwich, salad, sushi)<br>☐ Meal within sit down restaurant<br>☐ Other (please specify briefly) ____________.	$11.50
**# 2**<br>Receipt attached:<br>☑ Yes<br>☐ No	Store name: Coles Supermarket<br>Street number:<br>Street name/location: Moonee Ponds Central Shopping Centre<br>Suburb: Moonee Ponds	☐ Home<br>☑ Work<br>☐ Other (please specify) ____________.	☑ Car<br>☐ Public transport<br>☐ Walk/cycle<br>☐ Other<br>**OR**<br>☐ Home delivery	☑ Fresh fruit<br>☑ Fresh vegetables<br>☑ Grocery items (e.g. bread, milk, pasta, meat)<br>☐ Snack food (e.g. chips, chocolate)<br>☑ Soft drink<br>☐ Bottled water<br>☐ Hot fast food/takeaway (e.g. KFC, Chinese, pizza)<br>☐ Cold takeaway (e.g. sandwich, salad, sushi)<br>☐ Meal within sit down restaurant<br>☐ Other (please specify briefly) ____________.	$78.65

EXAMPLE

Day 1 continued

**Food purchase (FP) number/ receipt attached**<br><br>*(report for each different store visited)*	**What of the name of store where the food was purchased and address**<br><br>(<u>minimum information</u>: store name, street, suburb)	**Where were you prior to making this food purchase?**	**Primary mode of travel from the prior location to the food store.**	**What food was purchased**<br><br>Please keep and return receipt and note which day and food purchase number it relates to<br>**OR**<br>Fill in checklist below	**Amount spent $**
**# 3**<br><br>Receipt attached:<br>☑ Yes<br>☐ No	Store name: BP<br><br>Street number: 795<br><br>Street name/location: Mount Alexander Rd<br><br>Suburb: Moonee Ponds	☐ Home<br>☐ Work<br>☑ Other (please specify)<br>Supermarket	☑ Car<br>☐ Public transport<br>☐ Walk/cycle<br>☐ Other<br>**OR**<br>☐ Home delivery	☐ Fresh fruit<br>☐ Fresh vegetables<br>☐ Grocery items (e.g. bread, milk, pasta)<br>☑ Snack food (e.g. chips, chocolate)<br>☐ Soft drink<br>☐ Bottled water<br>☐ Hot fast food/takeaway (e.g. KFC, Chinese, pizza)<br>☐ Cold takeaway (e.g. sandwich, salad, sushi)<br>☐ Meal within sit down restaurant<br>☑ Other (please specify briefly) Big M	$6.30
**# 4**<br><br>Receipt attached:<br>☐ Yes<br>☑ No	Store name: Mario's Pizza<br><br>Street number:<br><br>Street name/location: Keilor Rd<br><br>Suburb: Niddrie	☑ Home<br>☐ Work<br>☐ Other (please specify)<br>__________.	☐ Car<br>☐ Public transport<br>☐ Walk/cycle<br>☐ Other<br>**OR**<br>☑ Home delivery	☐ Fresh fruit<br>☐ Fresh vegetables<br>☐ Grocery items (e.g. bread, milk, pasta)<br>☐ Snack food (e.g. chips, chocolate)<br>☑ Soft drink<br>☐ Bottled water<br>☑ Hot fast food/takeaway (e.g. KFC, Chinese, pizza)<br>☐ Cold takeaway (e.g. sandwich, salad, sushi)<br>☐ Meal within sit down restaurant<br>☐ Other (please specify briefly) __________.	$18

EXAMPLE

From the above food diary example, here are the relevant scenarios.

Food purchase 1

This food purchase occurred whilst the participant at work at Deakin University. They walked from their office to the café that was on campus to purchase a sandwich and bottle of water. They spent $11.50 on this purchase and did not receive a receipt.

Food purchase 2

This person drove from work straight to the supermarket in their home suburb, Moonee Ponds. Here they purchased a range of grocery items including pasta, milk, minced beef, lemonade, and a range of fresh fruit and vegetables. They spent a total of $78.65 during this purchase and keep the receipt which they marked as Day 1, FP #2. The food purchased at the supermarkets were used by the participants for lunch and dinner on Day 2 meaning no food purchases were made on Day 2.

Food purchase 3

On the way from the supermarket to home, the participant stopped at their local BP service station to put petrol in their car. Whilst inside the store they bought a chocolate bar and a Big M which cost $6.30. A receipt was acquired and was marked Day 1, FP #3.

Food purchase 4

That evening the participant ordered a pizza for dinner for themselves and partner. The pizza was home delivered with a bottle of Pepsi Max. The order cost $18 but no receipt was obtained.

THE FOLLOWING PAGES CONTAIN THE FOOD PURCHASING DIARY FOR YOU TO COMPLETE

EXAMPLE

FOOD PURCHASING DIARY

Day 1: ________________ **No food purchase made on this day:** ☐

**Food purchase (FP) number/ receipt attached**<br>*(report for each different store visited)*	**Store name/address**<br>(minimum information: store name, street/location, suburb)	**Where were you prior to making this food purchase?**	**Primary mode of travel from the prior location to the food store.**	**What food was purchased**<br>Please keep and return receipt and note which day and food purchase number it relates to<br>**OR**<br>Fill in checklist below	**Amount spent $**
**# 1**<br>Receipt attached:<br>☐ Yes<br>☐ No	Store name:<br>Street number:<br>Street name/location:<br>Suburb:	☐ Home<br>☐ Work<br>☐ Other (please specify)<br>________________________.	☐ Car<br>☐ Public transport<br>☐ Walk/cycle<br>☐ Other<br>**OR**<br>☐ Home delivery	☐ Fresh fruit<br>☐ Fresh vegetables<br>☐ Grocery items (e.g. bread, milk, pasta)<br>☐ Snack food (e.g. chips, chocolate)<br>☐ Soft drink<br>☐ Bottled water<br>☐ Hot fast food/takeaway (e.g. KFC, Chinese, pizza)<br>☐ Cold takeaway (e.g. sandwich, salad, sushi)<br>☐ Meal within sit down restaurant<br>☐ Other (please specify briefly) ____________________.	
**# 2**<br>Receipt attached:<br>☐ Yes<br>☐ No	Store name:<br>Street number:<br>Street name/location:<br>Suburb:	☐ Home<br>☐ Work<br>☐ Other (please specify)<br>________________________.	☐ Car<br>☐ Public transport<br>☐ Walk/cycle<br>☐ Other<br>**OR**<br>☐ Home delivery	☐ Fresh fruit<br>☐ Fresh vegetables<br>☐ Grocery items (e.g. bread, milk, pasta, meat)<br>☐ Snack food (e.g. chips, chocolate)<br>☐ Soft drink<br>☐ Bottled water<br>☐ Hot fast food/takeaway (e.g. KFC, Chinese, pizza)<br>☐ Cold takeaway (e.g. sandwich, salad, sushi)<br>☐ Meal within sit down restaurant<br>☐ Other (please specify briefly) ____________________.	

FOOD PURCHASING DIARY

Day 1 continued

**Food purchase (FP) number/ receipt attached**<br><br>*(report for each different store visited)*	**Store name/address**<br><br>(<u>minimum information:</u> store name, street/location, suburb)	**Where were you prior to making this food purchase?**	**Primary mode of travel from the prior location to the food store.**	**What food was purchased**<br><br>Please keep and return receipt and note which day and food purchase number it relates to<br>**OR**<br>Fill in checklist below	**Amount spent $**
**# 3**<br><br>Receipt attached:<br>☐ Yes<br>☐ No	Store name:<br><br>Street number:<br><br>Street name/location:<br><br>Suburb:	☐ Home<br>☐ Work<br>☐ Other (please specify)<br>____________________.	☐ Car<br>☐ Public transport<br>☐ Walk/cycle<br>☐ Other<br>**OR**<br>☐ Home delivery	☐ Fresh fruit<br>☐ Fresh vegetables<br>☐ Grocery items (e.g. bread, milk, pasta)<br>☐ Snack food (e.g. chips, chocolate)<br>☐ Soft drink<br>☐ Bottled water<br>☐ Hot fast food/takeaway (e.g. KFC, Chinese, pizza)<br>☐ Cold takeaway (e.g. sandwich, salad, sushi)<br>☐ Meal within sit down restaurant<br>☐ Other (please specify briefly) ________________.	
**# 4**<br><br>Receipt attached:<br>☐ Yes<br>☐ No	Store name:<br><br>Street number:<br><br>Street name/location:<br><br>Suburb:	☐ Home<br>☐ Work<br>☐ Other (please specify)<br>____________________.	☐ Car<br>☐ Public transport<br>☐ Walk/cycle<br>☐ Other<br>**OR**<br>☐ Home delivery	☐ Fresh fruit<br>☐ Fresh vegetables<br>☐ Grocery items (e.g. bread, milk, pasta, meat)<br>☐ Snack food (e.g. chips, chocolate)<br>☐ Soft drink<br>☐ Bottled water<br>☐ Hot fast food/takeaway (e.g. KFC, Chinese, pizza)<br>☐ Cold takeaway (e.g. sandwich, salad, sushi)<br>☐ Meal within sit down restaurant<br>☐ Other (please specify briefly) ________________.	

FOOD PURCHASING DIARY

Day 2: ________________ **No food purchase made on this day:** ☐

**Food purchase (FP) number/ receipt attached**<br><br>*(report for each different store visited)*	**Store name/address**<br><br>(<u>minimum information:</u> store name, street/location, suburb)	**Where were you prior to making this food purchase?**	**Primary mode of travel from the prior location to the food store.**	**What food was purchased**<br><br>Please keep and return receipt and note which day and food purchase number it relates to<br>**OR**<br>Fill in checklist below	**Amount spent $**
**# 1**<br><br>Receipt attached:<br>☐ Yes<br>☐ No	Store name:<br><br>Street number:<br><br>Street name/location:<br><br>Suburb:	☐ Home<br>☐ Work<br>☐ Other (please specify)<br>________________________.	☐ Car<br>☐ Public transport<br>☐ Walk/cycle<br>☐ Other<br>**OR**<br>☐ Home delivery	☐ Fresh fruit<br>☐ Fresh vegetables<br>☐ Grocery items (e.g. bread, milk, pasta)<br>☐ Snack food (e.g. chips, chocolate)<br>☐ Soft drink<br>☐ Bottled water<br>☐ Hot fast food/takeaway (e.g. KFC, Chinese, pizza)<br>☐ Cold takeaway (e.g. sandwich, salad, sushi)<br>☐ Meal within sit down restaurant<br>☐ Other (please specify briefly) ___________________.	
**# 2**<br><br>Receipt attached:<br>☐ Yes<br>☐ No	Store name:<br><br>Street number:<br><br>Street name/location:<br><br>Suburb:	☐ Home<br>☐ Work<br>☐ Other (please specify)<br>________________________.	☐ Car<br>☐ Public transport<br>☐ Walk/cycle<br>☐ Other<br>**OR**<br>☐ Home delivery	☐ Fresh fruit<br>☐ Fresh vegetables<br>☐ Grocery items (e.g. bread, milk, pasta, meat)<br>☐ Snack food (e.g. chips, chocolate)<br>☐ Soft drink<br>☐ Bottled water<br>☐ Hot fast food/takeaway (e.g. KFC, Chinese, pizza)<br>☐ Cold takeaway (e.g. sandwich, salad, sushi)<br>☐ Meal within sit down restaurant<br>☐ Other (please specify briefly) ___________________.	

FOOD PURCHASING DIARY

Day 2 continued

**Food purchase (FP) number/ receipt attached**<br><br>*(report for each different store visited)*	**Store name/address**<br><br>(minimum information: store name, street/location, suburb)	**Where were you prior to making this food purchase?**	**Primary mode of travel from the prior location to the food store.**	**What food was purchased**<br><br>Please keep and return receipt and note which day and food purchase number it relates to<br>**OR**<br>Fill in checklist below	**Amount spent $**
**# 3**<br><br>Receipt attached:<br>☐ Yes<br>☐ No	Store name:<br><br>Street number:<br><br>Street name/location:<br><br>Suburb:	☐ Home<br>☐ Work<br>☐ Other (please specify)<br>____________________.	☐ Car<br>☐ Public transport<br>☐ Walk/cycle<br>☐ Other<br>**OR**<br>☐ Home delivery	☐ Fresh fruit<br>☐ Fresh vegetables<br>☐ Grocery items (e.g. bread, milk, pasta)<br>☐ Snack food (e.g. chips, chocolate)<br>☐ Soft drink<br>☐ Bottled water<br>☐ Hot fast food/takeaway (e.g. KFC, Chinese, pizza)<br>☐ Cold takeaway (e.g. sandwich, salad, sushi)<br>☐ Meal within sit down restaurant<br>☐ Other (please specify briefly) ________________.	
**# 4**<br><br>Receipt attached:<br>☐ Yes<br>☐ No	Store name:<br><br>Street number:<br><br>Street name/location:<br><br>Suburb:	☐ Home<br>☐ Work<br>☐ Other (please specify)<br>____________________.	☐ Car<br>☐ Public transport<br>☐ Walk/cycle<br>☐ Other<br>**OR**<br>☐ Home delivery	☐ Fresh fruit<br>☐ Fresh vegetables<br>☐ Grocery items (e.g. bread, milk, pasta, meat)<br>☐ Snack food (e.g. chips, chocolate)<br>☐ Soft drink<br>☐ Bottled water<br>☐ Hot fast food/takeaway (e.g. KFC, Chinese, pizza)<br>☐ Cold takeaway (e.g. sandwich, salad, sushi)<br>☐ Meal within sit down restaurant<br>☐ Other (please specify briefly) ________________.	

FOOD PURCHASING DIARY

Day 3: ________________ **No food purchase made on this day:** ☐

**Food purchase (FP) number/ receipt attached**<br><br>*(report for each different store visited)*	**Store name/address**<br><br>(minimum information: store name, street/location, suburb)	**Where were you prior to making this food purchase?**	**Primary mode of travel from the prior location to the food store.**	**What food was purchased**<br><br>Please keep and return receipt and note which day and food purchase number it relates to<br>**OR**<br>Fill in checklist below	**Amount spent $**
**# 1**<br><br>Receipt attached:<br>☐ Yes<br>☐ No	Store name:<br><br>Street number:<br><br>Street name/location:<br><br>Suburb:	☐ Home<br>☐ Work<br>☐ Other (please specify)<br>________________________.	☐ Car<br>☐ Public transport<br>☐ Walk/cycle<br>☐ Other<br>**OR**<br>☐ Home delivery	☐ Fresh fruit<br>☐ Fresh vegetables<br>☐ Grocery items (e.g. bread, milk, pasta)<br>☐ Snack food (e.g. chips, chocolate)<br>☐ Soft drink<br>☐ Bottled water<br>☐ Hot fast food/takeaway (e.g. KFC, Chinese, pizza)<br>☐ Cold takeaway (e.g. sandwich, salad, sushi)<br>☐ Meal within sit down restaurant<br>☐ Other (please specify briefly) ____________________.	
**# 2**<br><br>Receipt attached:<br>☐ Yes<br>☐ No	Store name:<br><br>Street number:<br><br>Street name/location:<br><br>Suburb:	☐ Home<br>☐ Work<br>☐ Other (please specify)<br>________________________.	☐ Car<br>☐ Public transport<br>☐ Walk/cycle<br>☐ Other<br>**OR**<br>☐ Home delivery	☐ Fresh fruit<br>☐ Fresh vegetables<br>☐ Grocery items (e.g. bread, milk, pasta, meat)<br>☐ Snack food (e.g. chips, chocolate)<br>☐ Soft drink<br>☐ Bottled water<br>☐ Hot fast food/takeaway (e.g. KFC, Chinese, pizza)<br>☐ Cold takeaway (e.g. sandwich, salad, sushi)<br>☐ Meal within sit down restaurant<br>☐ Other (please specify briefly) ____________________.	

FOOD PURCHASING DIARY

Day 3 continued

**Food purchase (FP) number/ receipt attached**<br><br>*(report for each different store visited)*	**Store name/address**<br><br>(minimum information: store name, street/location, suburb)	**Where were you prior to making this food purchase?**	**Primary mode of travel from the prior location to the food store.**	**What food was purchased**<br><br>Please keep and return receipt and note which day and food purchase number it relates to<br>**OR**<br>Fill in checklist below	**Amount spent $**
**# 3**<br><br>Receipt attached:<br>☐ Yes<br>☐ No	Store name:<br><br>Street number:<br><br>Street name/location:<br><br>Suburb:	☐ Home<br>☐ Work<br>☐ Other (please specify)<br>______________________.	☐ Car<br>☐ Public transport<br>☐ Walk/cycle<br>☐ Other<br>**OR**<br>☐ Home delivery	☐ Fresh fruit<br>☐ Fresh vegetables<br>☐ Grocery items (e.g. bread, milk, pasta)<br>☐ Snack food (e.g. chips, chocolate)<br>☐ Soft drink<br>☐ Bottled water<br>☐ Hot fast food/takeaway (e.g. KFC, Chinese, pizza)<br>☐ Cold takeaway (e.g. sandwich, salad, sushi)<br>☐ Meal within sit down restaurant<br>☐ Other (please specify briefly) ______________.	
**# 4**<br><br>Receipt attached:<br>☐ Yes<br>☐ No	Store name:<br><br>Street number:<br><br>Street name/location:<br><br>Suburb:	☐ Home<br>☐ Work<br>☐ Other (please specify)<br>______________________.	☐ Car<br>☐ Public transport<br>☐ Walk/cycle<br>☐ Other<br>**OR**<br>☐ Home delivery	☐ Fresh fruit<br>☐ Fresh vegetables<br>☐ Grocery items (e.g. bread, milk, pasta, meat)<br>☐ Snack food (e.g. chips, chocolate)<br>☐ Soft drink<br>☐ Bottled water<br>☐ Hot fast food/takeaway (e.g. KFC, Chinese, pizza)<br>☐ Cold takeaway (e.g. sandwich, salad, sushi)<br>☐ Meal within sit down restaurant<br>☐ Other (please specify briefly) ______________.	

FOOD PURCHASING DIARY

Day 4: ________________ **No food purchase made on this day:** ☐

**Food purchase (FP) number/ receipt attached**<br><br>*(report for each different store visited)*	**Store name/address**<br><br>(<u>minimum information:</u> store name, street/location, suburb)	**Where were you prior to making this food purchase?**	**Primary mode of travel from the prior location to the food store.**	**What food was purchased**<br><br>Please keep and return receipt and note which day and food purchase number it relates to<br>**OR**<br>Fill in checklist below	**Amount spent $**
**# 1**<br><br>Receipt attached:<br>☐ Yes<br>☐ No	Store name:<br><br>Street number:<br><br>Street name/location:<br><br>Suburb:	☐ Home<br>☐ Work<br>☐ Other (please specify)<br>________________________.	☐ Car<br>☐ Public transport<br>☐ Walk/cycle<br>☐ Other<br>**OR**<br>☐ Home delivery	☐ Fresh fruit<br>☐ Fresh vegetables<br>☐ Grocery items (e.g. bread, milk, pasta)<br>☐ Snack food (e.g. chips, chocolate)<br>☐ Soft drink<br>☐ Bottled water<br>☐ Hot fast food/takeaway (e.g. KFC, Chinese, pizza)<br>☐ Cold takeaway (e.g. sandwich, salad, sushi)<br>☐ Meal within sit down restaurant<br>☐ Other (please specify briefly) ____________________.	
**# 2**<br><br>Receipt attached:<br>☐ Yes<br>☐ No	Store name:<br><br>Street number:<br><br>Street name/location:<br><br>Suburb:	☐ Home<br>☐ Work<br>☐ Other (please specify)<br>________________________.	☐ Car<br>☐ Public transport<br>☐ Walk/cycle<br>☐ Other<br>**OR**<br>☐ Home delivery	☐ Fresh fruit<br>☐ Fresh vegetables<br>☐ Grocery items (e.g. bread, milk, pasta, meat)<br>☐ Snack food (e.g. chips, chocolate)<br>☐ Soft drink<br>☐ Bottled water<br>☐ Hot fast food/takeaway (e.g. KFC, Chinese, pizza)<br>☐ Cold takeaway (e.g. sandwich, salad, sushi)<br>☐ Meal within sit down restaurant<br>☐ Other (please specify briefly) ____________________.	

FOOD PURCHASING DIARY

Day 4 continued

**Food purchase (FP) number/ receipt attached**<br><br>*(report for each different store visited)*	**Store name/address**<br><br>(minimum information: store name, street/location, suburb)	**Where were you prior to making this food purchase?**	**Primary mode of travel from the prior location to the food store.**	**What food was purchased**<br><br>Please keep and return receipt and note which day and food purchase number it relates to<br>**OR**<br>Fill in checklist below	**Amount spent $**
**# 3**<br><br>Receipt attached:<br>☐ Yes<br>☐ No	Store name:<br><br>Street number:<br><br>Street name/location:<br><br>Suburb:	☐ Home<br>☐ Work<br>☐ Other (please specify)<br>________________________.	☐ Car<br>☐ Public transport<br>☐ Walk/cycle<br>☐ Other<br>**OR**<br>☐ Home delivery	☐ Fresh fruit<br>☐ Fresh vegetables<br>☐ Grocery items (e.g. bread, milk, pasta)<br>☐ Snack food (e.g. chips, chocolate)<br>☐ Soft drink<br>☐ Bottled water<br>☐ Hot fast food/takeaway (e.g. KFC, Chinese, pizza)<br>☐ Cold takeaway (e.g. sandwich, salad, sushi)<br>☐ Meal within sit down restaurant<br>☐ Other (please specify briefly) ____________________.	
**# 4**<br><br>Receipt attached:<br>☐ Yes<br>☐ No	Store name:<br><br>Street number:<br><br>Street name/location:<br><br>Suburb:	☐ Home<br>☐ Work<br>☐ Other (please specify)<br>________________________.	☐ Car<br>☐ Public transport<br>☐ Walk/cycle<br>☐ Other<br>**OR**<br>☐ Home delivery	☐ Fresh fruit<br>☐ Fresh vegetables<br>☐ Grocery items (e.g. bread, milk, pasta, meat)<br>☐ Snack food (e.g. chips, chocolate)<br>☐ Soft drink<br>☐ Bottled water<br>☐ Hot fast food/takeaway (e.g. KFC, Chinese, pizza)<br>☐ Cold takeaway (e.g. sandwich, salad, sushi)<br>☐ Meal within sit down restaurant<br>☐ Other (please specify briefly) ____________________.	

FOOD PURCHASING DIARY

Day 5: ________________ **No food purchase made on this day:** ☐

**Food purchase (FP) number/ receipt attached**<br><br>*(report for each different store visited)*	**Store name/address**<br><br>(<u>minimum information:</u> store name, street/location, suburb)	**Where were you prior to making this food purchase?**	**Primary mode of travel from the prior location to the food store.**	**What food was purchased**<br><br>Please keep and return receipt and note which day and food purchase number it relates to<br>**OR**<br>Fill in checklist below	**Amount spent $**
**# 1**<br><br>Receipt attached:<br>☐ Yes<br>☐ No	Store name:<br><br>Street number:<br><br>Street name/location:<br><br>Suburb:	☐ Home<br>☐ Work<br>☐ Other (please specify)<br>________________________.	☐ Car<br>☐ Public transport<br>☐ Walk/cycle<br>☐ Other<br>**OR**<br>☐ Home delivery	☐ Fresh fruit<br>☐ Fresh vegetables<br>☐ Grocery items (e.g. bread, milk, pasta)<br>☐ Snack food (e.g. chips, chocolate)<br>☐ Soft drink<br>☐ Bottled water<br>☐ Hot fast food/takeaway (e.g. KFC, Chinese, pizza)<br>☐ Cold takeaway (e.g. sandwich, salad, sushi)<br>☐ Meal within sit down restaurant<br>☐ Other (please specify briefly) ____________________.	
**# 2**<br><br>Receipt attached:<br>☐ Yes<br>☐ No	Store name:<br><br>Street number:<br><br>Street name/location:<br><br>Suburb:	☐ Home<br>☐ Work<br>☐ Other (please specify)<br>________________________.	☐ Car<br>☐ Public transport<br>☐ Walk/cycle<br>☐ Other<br>**OR**<br>☐ Home delivery	☐ Fresh fruit<br>☐ Fresh vegetables<br>☐ Grocery items (e.g. bread, milk, pasta, meat)<br>☐ Snack food (e.g. chips, chocolate)<br>☐ Soft drink<br>☐ Bottled water<br>☐ Hot fast food/takeaway (e.g. KFC, Chinese, pizza)<br>☐ Cold takeaway (e.g. sandwich, salad, sushi)<br>☐ Meal within sit down restaurant<br>☐ Other (please specify briefly) ____________________.	

FOOD PURCHASING DIARY

Day 5 continued

**Food purchase (FP) number/ receipt attached**<br><br>*(report for each different store visited)*	**Store name/address**<br><br>(minimum information: store name, street/location, suburb)	**Where were you prior to making this food purchase?**	**Primary mode of travel from the prior location to the food store.**	**What food was purchased**<br><br>Please keep and return receipt and note which day and food purchase number it relates to<br>**OR**<br>Fill in checklist below	**Amount spent $**
**# 3**<br><br>Receipt attached:<br>☐ Yes<br>☐ No	Store name:<br><br>Street number:<br><br>Street name/location:<br><br>Suburb:	☐ Home<br>☐ Work<br>☐ Other (please specify)<br>____________________.	☐ Car<br>☐ Public transport<br>☐ Walk/cycle<br>☐ Other<br>**OR**<br>☐ Home delivery	☐ Fresh fruit<br>☐ Fresh vegetables<br>☐ Grocery items (e.g. bread, milk, pasta)<br>☐ Snack food (e.g. chips, chocolate)<br>☐ Soft drink<br>☐ Bottled water<br>☐ Hot fast food/takeaway (e.g. KFC, Chinese, pizza)<br>☐ Cold takeaway (e.g. sandwich, salad, sushi)<br>☐ Meal within sit down restaurant<br>☐ Other (please specify briefly) ________________.	
**# 4**<br><br>Receipt attached:<br>☐ Yes<br>☐ No	Store name:<br><br>Street number:<br><br>Street name/location:<br><br>Suburb:	☐ Home<br>☐ Work<br>☐ Other (please specify)<br>____________________.	☐ Car<br>☐ Public transport<br>☐ Walk/cycle<br>☐ Other<br>**OR**<br>☐ Home delivery	☐ Fresh fruit<br>☐ Fresh vegetables<br>☐ Grocery items (e.g. bread, milk, pasta, meat)<br>☐ Snack food (e.g. chips, chocolate)<br>☐ Soft drink<br>☐ Bottled water<br>☐ Hot fast food/takeaway (e.g. KFC, Chinese, pizza)<br>☐ Cold takeaway (e.g. sandwich, salad, sushi)<br>☐ Meal within sit down restaurant<br>☐ Other (please specify briefly) ________________.	

FOOD PURCHASING DIARY

Day 6: ________________ **No food purchase made on this day:** ☐

**Food purchase (FP) number/ receipt attached**<br><br>*(report for each different store visited)*	**Store name/address**<br><br>(<u>minimum information:</u> store name, street/location, suburb)	**Where were you prior to making this food purchase?**	**Primary mode of travel from the prior location to the food store.**	**What food was purchased**<br><br>Please keep and return receipt and note which day and food purchase number it relates to<br>**OR**<br>Fill in checklist below	**Amount spent $**
**# 1**<br><br>Receipt attached:<br>☐ Yes<br>☐ No	Store name:<br><br>Street number:<br><br>Street name/location:<br><br>Suburb:	☐ Home<br>☐ Work<br>☐ Other (please specify)<br>________________________.	☐ Car<br>☐ Public transport<br>☐ Walk/cycle<br>☐ Other<br>**OR**<br>☐ Home delivery	☐ Fresh fruit<br>☐ Fresh vegetables<br>☐ Grocery items (e.g. bread, milk, pasta)<br>☐ Snack food (e.g. chips, chocolate)<br>☐ Soft drink<br>☐ Bottled water<br>☐ Hot fast food/takeaway (e.g. KFC, Chinese, pizza)<br>☐ Cold takeaway (e.g. sandwich, salad, sushi)<br>☐ Meal within sit down restaurant<br>☐ Other (please specify briefly) ____________________.	
**# 2**<br><br>Receipt attached:<br>☐ Yes<br>☐ No	Store name:<br><br>Street number:<br><br>Street name/location:<br><br>Suburb:	☐ Home<br>☐ Work<br>☐ Other (please specify)<br>________________________.	☐ Car<br>☐ Public transport<br>☐ Walk/cycle<br>☐ Other<br>**OR**<br>☐ Home delivery	☐ Fresh fruit<br>☐ Fresh vegetables<br>☐ Grocery items (e.g. bread, milk, pasta, meat)<br>☐ Snack food (e.g. chips, chocolate)<br>☐ Soft drink<br>☐ Bottled water<br>☐ Hot fast food/takeaway (e.g. KFC, Chinese, pizza)<br>☐ Cold takeaway (e.g. sandwich, salad, sushi)<br>☐ Meal within sit down restaurant<br>☐ Other (please specify briefly) ____________________.	

FOOD PURCHASING DIARY

Day 6 continued

**Food purchase (FP) number/ receipt attached**<br><br>*(report for each different store visited)*	**Store name/address**<br><br>(<u>minimum information:</u> store name, street/location, suburb)	**Where were you prior to making this food purchase?**	**Primary mode of travel from the prior location to the food store.**	**What food was purchased**<br><br>Please keep and return receipt and note which day and food purchase number it relates to<br>**OR**<br>Fill in checklist below	**Amount spent $**
**# 3**<br><br>Receipt attached:<br>☐ Yes<br>☐ No	Store name:<br><br>Street number:<br><br>Street name/location:<br><br>Suburb:	☐ Home<br>☐ Work<br>☐ Other (please specify)<br>____________________.	☐ Car<br>☐ Public transport<br>☐ Walk/cycle<br>☐ Other<br>**OR**<br>☐ Home delivery	☐ Fresh fruit<br>☐ Fresh vegetables<br>☐ Grocery items (e.g. bread, milk, pasta)<br>☐ Snack food (e.g. chips, chocolate)<br>☐ Soft drink<br>☐ Bottled water<br>☐ Hot fast food/takeaway (e.g. KFC, Chinese, pizza)<br>☐ Cold takeaway (e.g. sandwich, salad, sushi)<br>☐ Meal within sit down restaurant<br>☐ Other (please specify briefly) ________________.	
**# 4**<br><br>Receipt attached:<br>☐ Yes<br>☐ No	Store name:<br><br>Street number:<br><br>Street name/location:<br><br>Suburb:	☐ Home<br>☐ Work<br>☐ Other (please specify)<br>____________________.	☐ Car<br>☐ Public transport<br>☐ Walk/cycle<br>☐ Other<br>**OR**<br>☐ Home delivery	☐ Fresh fruit<br>☐ Fresh vegetables<br>☐ Grocery items (e.g. bread, milk, pasta, meat)<br>☐ Snack food (e.g. chips, chocolate)<br>☐ Soft drink<br>☐ Bottled water<br>☐ Hot fast food/takeaway (e.g. KFC, Chinese, pizza)<br>☐ Cold takeaway (e.g. sandwich, salad, sushi)<br>☐ Meal within sit down restaurant<br>☐ Other (please specify briefly) ________________.	

FOOD PURCHASING DIARY

Day 7: ________________ **No food purchase made on this day:** ☐

**Food purchase (FP) number/ receipt attached**<br><br>*(report for each different store visited)*	**Store name/address**<br><br>(<u>minimum information:</u> store name, street/location, suburb)	**Where were you prior to making this food purchase?**	**Primary mode of travel from the prior location to the food store.**	**What food was purchased**<br><br>Please keep and return receipt and note which day and food purchase number it relates to<br>**OR**<br>Fill in checklist below	**Amount spent $**
**# 1**<br><br>Receipt attached:<br>☐ Yes<br>☐ No	Store name:<br><br>Street number:<br><br>Street name/location:<br><br>Suburb:	☐ Home<br>☐ Work<br>☐ Other (please specify)<br>________________________.	☐ Car<br>☐ Public transport<br>☐ Walk/cycle<br>☐ Other<br>**OR**<br>☐ Home delivery	☐ Fresh fruit<br>☐ Fresh vegetables<br>☐ Grocery items (e.g. bread, milk, pasta)<br>☐ Snack food (e.g. chips, chocolate)<br>☐ Soft drink<br>☐ Bottled water<br>☐ Hot fast food/takeaway (e.g. KFC, Chinese, pizza)<br>☐ Cold takeaway (e.g. sandwich, salad, sushi)<br>☐ Meal within sit down restaurant<br>☐ Other (please specify briefly) ____________________.	
**# 2**<br><br>Receipt attached:<br>☐ Yes<br>☐ No	Store name:<br><br>Street number:<br><br>Street name/location:<br><br>Suburb:	☐ Home<br>☐ Work<br>☐ Other (please specify)<br>________________________.	☐ Car<br>☐ Public transport<br>☐ Walk/cycle<br>☐ Other<br>**OR**<br>☐ Home delivery	☐ Fresh fruit<br>☐ Fresh vegetables<br>☐ Grocery items (e.g. bread, milk, pasta, meat)<br>☐ Snack food (e.g. chips, chocolate)<br>☐ Soft drink<br>☐ Bottled water<br>☐ Hot fast food/takeaway (e.g. KFC, Chinese, pizza)<br>☐ Cold takeaway (e.g. sandwich, salad, sushi)<br>☐ Meal within sit down restaurant<br>☐ Other (please specify briefly) ____________________.	

FOOD PURCHASING DIARY

Day 7 continued

**Food purchase (FP) number/ receipt attached**<br><br>*(report for each different store visited)*	**Store name/address**<br><br>(<u>minimum information:</u> store name, street/location, suburb)	**Where were you prior to making this food purchase?**	**Primary mode of travel from the prior location to the food store.**	**What food was purchased**<br><br>Please keep and return receipt and note which day and food purchase number it relates to<br>**OR**<br>Fill in checklist below	**Amount spent $**
**# 3**<br><br>Receipt attached:<br>☐ Yes<br>☐ No	Store name:<br><br>Street number:<br><br>Street name/location:<br><br>Suburb:	☐ Home<br>☐ Work<br>☐ Other (please specify)<br>____________________.	☐ Car<br>☐ Public transport<br>☐ Walk/cycle<br>☐ Other<br>**OR**<br>☐ Home delivery	☐ Fresh fruit<br>☐ Fresh vegetables<br>☐ Grocery items (e.g. bread, milk, pasta)<br>☐ Snack food (e.g. chips, chocolate)<br>☐ Soft drink<br>☐ Bottled water<br>☐ Hot fast food/takeaway (e.g. KFC, Chinese, pizza)<br>☐ Cold takeaway (e.g. sandwich, salad, sushi)<br>☐ Meal within sit down restaurant<br>☐ Other (please specify briefly) ________________.	
**# 4**<br><br>Receipt attached:<br>☐ Yes<br>☐ No	Store name:<br><br>Street number:<br><br>Street name/location:<br><br>Suburb:	☐ Home<br>☐ Work<br>☐ Other (please specify)<br>____________________.	☐ Car<br>☐ Public transport<br>☐ Walk/cycle<br>☐ Other<br>**OR**<br>☐ Home delivery	☐ Fresh fruit<br>☐ Fresh vegetables<br>☐ Grocery items (e.g. bread, milk, pasta, meat)<br>☐ Snack food (e.g. chips, chocolate)<br>☐ Soft drink<br>☐ Bottled water<br>☐ Hot fast food/takeaway (e.g. KFC, Chinese, pizza)<br>☐ Cold takeaway (e.g. sandwich, salad, sushi)<br>☐ Meal within sit down restaurant<br>☐ Other (please specify briefly) ________________.	

FOOD PURCHASING DIARY

Day 8: ________________ **No food purchase made on this day:** ☐

**Food purchase (FP) number/ receipt attached**<br><br>*(report for each different store visited)*	**Store name/address**<br><br>(<u>minimum information:</u> store name, street/location, suburb)	**Where were you prior to making this food purchase?**	**Primary mode of travel from the prior location to the food store.**	**What food was purchased**<br><br>Please keep and return receipt and note which day and food purchase number it relates to<br>**OR**<br>Fill in checklist below	**Amount spent $**
**# 1**<br><br>Receipt attached:<br>☐ Yes<br>☐ No	Store name:<br><br>Street number:<br><br>Street name/location:<br><br>Suburb:	☐ Home<br>☐ Work<br>☐ Other (please specify)<br>________________________.	☐ Car<br>☐ Public transport<br>☐ Walk/cycle<br>☐ Other<br>**OR**<br>☐ Home delivery	☐ Fresh fruit<br>☐ Fresh vegetables<br>☐ Grocery items (e.g. bread, milk, pasta)<br>☐ Snack food (e.g. chips, chocolate)<br>☐ Soft drink<br>☐ Bottled water<br>☐ Hot fast food/takeaway (e.g. KFC, Chinese, pizza)<br>☐ Cold takeaway (e.g. sandwich, salad, sushi)<br>☐ Meal within sit down restaurant<br>☐ Other (please specify briefly) ____________________.	
**# 2**<br><br>Receipt attached:<br>☐ Yes<br>☐ No	Store name:<br><br>Street number:<br><br>Street name/location:<br><br>Suburb:	☐ Home<br>☐ Work<br>☐ Other (please specify)<br>________________________.	☐ Car<br>☐ Public transport<br>☐ Walk/cycle<br>☐ Other<br>**OR**<br>☐ Home delivery	☐ Fresh fruit<br>☐ Fresh vegetables<br>☐ Grocery items (e.g. bread, milk, pasta, meat)<br>☐ Snack food (e.g. chips, chocolate)<br>☐ Soft drink<br>☐ Bottled water<br>☐ Hot fast food/takeaway (e.g. KFC, Chinese, pizza)<br>☐ Cold takeaway (e.g. sandwich, salad, sushi)<br>☐ Meal within sit down restaurant<br>☐ Other (please specify briefly) ____________________.	

FOOD PURCHASING DIARY

Day 8 continued

**Food purchase (FP) number/ receipt attached**<br><br>*(report for each different store visited)*	**Store name/address**<br><br>(<u>minimum information:</u> store name, street/location, suburb)	**Where were you prior to making this food purchase?**	**Primary mode of travel from the prior location to the food store.**	**What food was purchased**<br><br>Please keep and return receipt and note which day and food purchase number it relates to<br>**OR**<br>Fill in checklist below	**Amount spent $**
**# 3**<br><br>Receipt attached:<br>☐ Yes<br>☐ No	Store name:<br><br>Street number:<br><br>Street name/location:<br><br>Suburb:	☐ Home<br>☐ Work<br>☐ Other (please specify)<br>____________________.	☐ Car<br>☐ Public transport<br>☐ Walk/cycle<br>☐ Other<br>**OR**<br>☐ Home delivery	☐ Fresh fruit<br>☐ Fresh vegetables<br>☐ Grocery items (e.g. bread, milk, pasta)<br>☐ Snack food (e.g. chips, chocolate)<br>☐ Soft drink<br>☐ Bottled water<br>☐ Hot fast food/takeaway (e.g. KFC, Chinese, pizza)<br>☐ Cold takeaway (e.g. sandwich, salad, sushi)<br>☐ Meal within sit down restaurant<br>☐ Other (please specify briefly) ________________.	
**# 4**<br><br>Receipt attached:<br>☐ Yes<br>☐ No	Store name:<br><br>Street number:<br><br>Street name/location:<br><br>Suburb:	☐ Home<br>☐ Work<br>☐ Other (please specify)<br>____________________.	☐ Car<br>☐ Public transport<br>☐ Walk/cycle<br>☐ Other<br>**OR**<br>☐ Home delivery	☐ Fresh fruit<br>☐ Fresh vegetables<br>☐ Grocery items (e.g. bread, milk, pasta, meat)<br>☐ Snack food (e.g. chips, chocolate)<br>☐ Soft drink<br>☐ Bottled water<br>☐ Hot fast food/takeaway (e.g. KFC, Chinese, pizza)<br>☐ Cold takeaway (e.g. sandwich, salad, sushi)<br>☐ Meal within sit down restaurant<br>☐ Other (please specify briefly) ________________.	

FOOD PURCHASING DIARY

Day 9: ________________ **No food purchase made on this day:** ☐

**Food purchase (FP) number/ receipt attached**<br><br>*(report for each different store visited)*	**Store name/address**<br><br>(minimum information: store name, street/location, suburb)	**Where were you prior to making this food purchase?**	**Primary mode of travel from the prior location to the food store.**	**What food was purchased**<br><br>Please keep and return receipt and note which day and food purchase number it relates to<br>**OR**<br>Fill in checklist below	**Amount spent $**
**# 1**<br><br>Receipt attached:<br>☐ Yes<br>☐ No	Store name:<br><br>Street number:<br><br>Street name/location:<br><br>Suburb:	☐ Home<br>☐ Work<br>☐ Other (please specify)<br>________________________.	☐ Car<br>☐ Public transport<br>☐ Walk/cycle<br>☐ Other<br>**OR**<br>☐ Home delivery	☐ Fresh fruit<br>☐ Fresh vegetables<br>☐ Grocery items (e.g. bread, milk, pasta)<br>☐ Snack food (e.g. chips, chocolate)<br>☐ Soft drink<br>☐ Bottled water<br>☐ Hot fast food/takeaway (e.g. KFC, Chinese, pizza)<br>☐ Cold takeaway (e.g. sandwich, salad, sushi)<br>☐ Meal within sit down restaurant<br>☐ Other (please specify briefly) ____________________.	
**# 2**<br><br>Receipt attached:<br>☐ Yes<br>☐ No	Store name:<br><br>Street number:<br><br>Street name/location:<br><br>Suburb:	☐ Home<br>☐ Work<br>☐ Other (please specify)<br>________________________.	☐ Car<br>☐ Public transport<br>☐ Walk/cycle<br>☐ Other<br>**OR**<br>☐ Home delivery	☐ Fresh fruit<br>☐ Fresh vegetables<br>☐ Grocery items (e.g. bread, milk, pasta, meat)<br>☐ Snack food (e.g. chips, chocolate)<br>☐ Soft drink<br>☐ Bottled water<br>☐ Hot fast food/takeaway (e.g. KFC, Chinese, pizza)<br>☐ Cold takeaway (e.g. sandwich, salad, sushi)<br>☐ Meal within sit down restaurant<br>☐ Other (please specify briefly) ____________________.	

FOOD PURCHASING DIARY

Day 9 continued

**Food purchase (FP) number/ receipt attached**<br><br>*(report for each different store visited)*	**Store name/address**<br><br>(<u>minimum information:</u> store name, street/location, suburb)	**Where were you prior to making this food purchase?**	**Primary mode of travel from the prior location to the food store.**	**What food was purchased**<br><br>Please keep and return receipt and note which day and food purchase number it relates to<br>**OR**<br>Fill in checklist below	**Amount spent $**
**# 3**<br><br>Receipt attached:<br>☐ Yes<br>☐ No	Store name:<br><br>Street number:<br><br>Street name/location:<br><br>Suburb:	☐ Home<br>☐ Work<br>☐ Other (please specify)<br>____________________.	☐ Car<br>☐ Public transport<br>☐ Walk/cycle<br>☐ Other<br>**OR**<br>☐ Home delivery	☐ Fresh fruit<br>☐ Fresh vegetables<br>☐ Grocery items (e.g. bread, milk, pasta)<br>☐ Snack food (e.g. chips, chocolate)<br>☐ Soft drink<br>☐ Bottled water<br>☐ Hot fast food/takeaway (e.g. KFC, Chinese, pizza)<br>☐ Cold takeaway (e.g. sandwich, salad, sushi)<br>☐ Meal within sit down restaurant<br>☐ Other (please specify briefly) ________________.	
**# 4**<br><br>Receipt attached:<br>☐ Yes<br>☐ No	Store name:<br><br>Street number:<br><br>Street name/location:<br><br>Suburb:	☐ Home<br>☐ Work<br>☐ Other (please specify)<br>____________________.	☐ Car<br>☐ Public transport<br>☐ Walk/cycle<br>☐ Other<br>**OR**<br>☐ Home delivery	☐ Fresh fruit<br>☐ Fresh vegetables<br>☐ Grocery items (e.g. bread, milk, pasta, meat)<br>☐ Snack food (e.g. chips, chocolate)<br>☐ Soft drink<br>☐ Bottled water<br>☐ Hot fast food/takeaway (e.g. KFC, Chinese, pizza)<br>☐ Cold takeaway (e.g. sandwich, salad, sushi)<br>☐ Meal within sit down restaurant<br>☐ Other (please specify briefly) ________________.	

FOOD PURCHASING DIARY

Day 10: ______________________ **No food purchase made on this day:** ☐

**Food purchase (FP) number/ receipt attached**<br><br>*(report for each different store visited)*	**Store name/address**<br><br>(minimum information: store name, street/location, suburb)	**Where were you prior to making this food purchase?**	**Primary mode of travel from the prior location to the food store.**	**What food was purchased**<br><br>Please keep and return receipt and note which day and food purchase number it relates to<br>**OR**<br>Fill in checklist below	**Amount spent $**
**# 1**<br><br>Receipt attached:<br>☐ Yes<br>☐ No	Store name:<br><br>Street number:<br><br>Street name/location:<br><br>Suburb:	☐ Home<br>☐ Work<br>☐ Other (please specify)<br>______________________.	☐ Car<br>☐ Public transport<br>☐ Walk/cycle<br>☐ Other<br>**OR**<br>☐ Home delivery	☐ Fresh fruit<br>☐ Fresh vegetables<br>☐ Grocery items (e.g. bread, milk, pasta)<br>☐ Snack food (e.g. chips, chocolate)<br>☐ Soft drink<br>☐ Bottled water<br>☐ Hot fast food/takeaway (e.g. KFC, Chinese, pizza)<br>☐ Cold takeaway (e.g. sandwich, salad, sushi)<br>☐ Meal within sit down restaurant<br>☐ Other (please specify briefly) ________________.	
**# 2**<br><br>Receipt attached:<br>☐ Yes<br>☐ No	Store name:<br><br>Street number:<br><br>Street name/location:<br><br>Suburb:	☐ Home<br>☐ Work<br>☐ Other (please specify)<br>______________________.	☐ Car<br>☐ Public transport<br>☐ Walk/cycle<br>☐ Other<br>**OR**<br>☐ Home delivery	☐ Fresh fruit<br>☐ Fresh vegetables<br>☐ Grocery items (e.g. bread, milk, pasta, meat)<br>☐ Snack food (e.g. chips, chocolate)<br>☐ Soft drink<br>☐ Bottled water<br>☐ Hot fast food/takeaway (e.g. KFC, Chinese, pizza)<br>☐ Cold takeaway (e.g. sandwich, salad, sushi)<br>☐ Meal within sit down restaurant<br>☐ Other (please specify briefly) ________________.	

FOOD PURCHASING DIARY

Day 10 continued

**Food purchase (FP) number/ receipt attached**<br><br>*(report for each different store visited)*	**Store name/address**<br><br>(<u>minimum information:</u> store name, street/location, suburb)	**Where were you prior to making this food purchase?**	**Primary mode of travel from the prior location to the food store.**	**What food was purchased**<br><br>Please keep and return receipt and note which day and food purchase number it relates to<br>**OR**<br>Fill in checklist below	**Amount spent $**
**# 3**<br><br>Receipt attached:<br>☐ Yes<br>☐ No	Store name:<br><br>Street number:<br><br>Street name/location:<br><br>Suburb:	☐ Home<br>☐ Work<br>☐ Other (please specify)<br>________________________.	☐ Car<br>☐ Public transport<br>☐ Walk/cycle<br>☐ Other<br>**OR**<br>☐ Home delivery	☐ Fresh fruit<br>☐ Fresh vegetables<br>☐ Grocery items (e.g. bread, milk, pasta)<br>☐ Snack food (e.g. chips, chocolate)<br>☐ Soft drink<br>☐ Bottled water<br>☐ Hot fast food/takeaway (e.g. KFC, Chinese, pizza)<br>☐ Cold takeaway (e.g. sandwich, salad, sushi)<br>☐ Meal within sit down restaurant<br>☐ Other (please specify briefly) ____________________.	
**# 4**<br><br>Receipt attached:<br>☐ Yes<br>☐ No	Store name:<br><br>Street number:<br><br>Street name/location:<br><br>Suburb:	☐ Home<br>☐ Work<br>☐ Other (please specify)<br>________________________.	☐ Car<br>☐ Public transport<br>☐ Walk/cycle<br>☐ Other<br>**OR**<br>☐ Home delivery	☐ Fresh fruit<br>☐ Fresh vegetables<br>☐ Grocery items (e.g. bread, milk, pasta, meat)<br>☐ Snack food (e.g. chips, chocolate)<br>☐ Soft drink<br>☐ Bottled water<br>☐ Hot fast food/takeaway (e.g. KFC, Chinese, pizza)<br>☐ Cold takeaway (e.g. sandwich, salad, sushi)<br>☐ Meal within sit down restaurant<br>☐ Other (please specify briefly) ____________________.	

FOOD PURCHASING DIARY

Day 11: ______________________ **No food purchase made on this day:** ☐

**Food purchase (FP) number/ receipt attached**<br><br>*(report for each different store visited)*	**Store name/address**<br><br>(<u>minimum information:</u> store name, street/location, suburb)	**Where were you prior to making this food purchase?**	**Primary mode of travel from the prior location to the food store.**	**What food was purchased**<br><br>Please keep and return receipt and note which day and food purchase number it relates to<br>**OR**<br>Fill in checklist below	**Amount spent $**
**# 1**<br><br>Receipt attached:<br>☐ Yes<br>☐ No	Store name:<br><br>Street number:<br><br>Street name/location:<br><br>Suburb:	☐ Home<br>☐ Work<br>☐ Other (please specify)<br>______________________.	☐ Car<br>☐ Public transport<br>☐ Walk/cycle<br>☐ Other<br>**OR**<br>☐ Home delivery	☐ Fresh fruit<br>☐ Fresh vegetables<br>☐ Grocery items (e.g. bread, milk, pasta)<br>☐ Snack food (e.g. chips, chocolate)<br>☐ Soft drink<br>☐ Bottled water<br>☐ Hot fast food/takeaway (e.g. KFC, Chinese, pizza)<br>☐ Cold takeaway (e.g. sandwich, salad, sushi)<br>☐ Meal within sit down restaurant<br>☐ Other (please specify briefly) ______________.	
**# 2**<br><br>Receipt attached:<br>☐ Yes<br>☐ No	Store name:<br><br>Street number:<br><br>Street name/location:<br><br>Suburb:	☐ Home<br>☐ Work<br>☐ Other (please specify)<br>______________________.	☐ Car<br>☐ Public transport<br>☐ Walk/cycle<br>☐ Other<br>**OR**<br>☐ Home delivery	☐ Fresh fruit<br>☐ Fresh vegetables<br>☐ Grocery items (e.g. bread, milk, pasta, meat)<br>☐ Snack food (e.g. chips, chocolate)<br>☐ Soft drink<br>☐ Bottled water<br>☐ Hot fast food/takeaway (e.g. KFC, Chinese, pizza)<br>☐ Cold takeaway (e.g. sandwich, salad, sushi)<br>☐ Meal within sit down restaurant<br>☐ Other (please specify briefly) ______________.	

FOOD PURCHASING DIARY

Day 11 continued

**Food purchase (FP) number/ receipt attached**<br><br>*(report for each different store visited)*	**Store name/address**<br><br>(minimum information: store name, street/location, suburb)	**Where were you prior to making this food purchase?**	**Primary mode of travel from the prior location to the food store.**	**What food was purchased**<br><br>Please keep and return receipt and note which day and food purchase number it relates to<br>**OR**<br>Fill in checklist below	**Amount spent $**
**# 3**<br><br>Receipt attached:<br>☐ Yes<br>☐ No	Store name:<br><br>Street number:<br><br>Street name/location:<br><br>Suburb:	☐ Home<br>☐ Work<br>☐ Other (please specify)<br>________________.	☐ Car<br>☐ Public transport<br>☐ Walk/cycle<br>☐ Other<br>**OR**<br>☐ Home delivery	☐ Fresh fruit<br>☐ Fresh vegetables<br>☐ Grocery items (e.g. bread, milk, pasta)<br>☐ Snack food (e.g. chips, chocolate)<br>☐ Soft drink<br>☐ Bottled water<br>☐ Hot fast food/takeaway (e.g. KFC, Chinese, pizza)<br>☐ Cold takeaway (e.g. sandwich, salad, sushi)<br>☐ Meal within sit down restaurant<br>☐ Other (please specify briefly) ____________.	
**# 4**<br><br>Receipt attached:<br>☐ Yes<br>☐ No	Store name:<br><br>Street number:<br><br>Street name/location:<br><br>Suburb:	☐ Home<br>☐ Work<br>☐ Other (please specify)<br>________________.	☐ Car<br>☐ Public transport<br>☐ Walk/cycle<br>☐ Other<br>**OR**<br>☐ Home delivery	☐ Fresh fruit<br>☐ Fresh vegetables<br>☐ Grocery items (e.g. bread, milk, pasta, meat)<br>☐ Snack food (e.g. chips, chocolate)<br>☐ Soft drink<br>☐ Bottled water<br>☐ Hot fast food/takeaway (e.g. KFC, Chinese, pizza)<br>☐ Cold takeaway (e.g. sandwich, salad, sushi)<br>☐ Meal within sit down restaurant<br>☐ Other (please specify briefly) ____________.	

FOOD PURCHASING DIARY

Day 12: ______________________ **No food purchase made on this day:** ☐

**Food purchase (FP) number/ receipt attached**<br><br>*(report for each different store visited)*	**Store name/address**<br><br>(<u>minimum information:</u> store name, street/location, suburb)	**Where were you prior to making this food purchase?**	**Primary mode of travel from the prior location to the food store.**	**What food was purchased**<br><br>Please keep and return receipt and note which day and food purchase number it relates to<br>**OR**<br>Fill in checklist below	**Amount spent $**
**# 1**<br><br>Receipt attached:<br>☐ Yes<br>☐ No	Store name:<br><br>Street number:<br><br>Street name/location:<br><br>Suburb:	☐ Home<br>☐ Work<br>☐ Other (please specify)<br>________________________.	☐ Car<br>☐ Public transport<br>☐ Walk/cycle<br>☐ Other<br>**OR**<br>☐ Home delivery	☐ Fresh fruit<br>☐ Fresh vegetables<br>☐ Grocery items (e.g. bread, milk, pasta)<br>☐ Snack food (e.g. chips, chocolate)<br>☐ Soft drink<br>☐ Bottled water<br>☐ Hot fast food/takeaway (e.g. KFC, Chinese, pizza)<br>☐ Cold takeaway (e.g. sandwich, salad, sushi)<br>☐ Meal within sit down restaurant<br>☐ Other (please specify briefly) ____________________.	
**# 2**<br><br>Receipt attached:<br>☐ Yes<br>☐ No	Store name:<br><br>Street number:<br><br>Street name/location:<br><br>Suburb:	☐ Home<br>☐ Work<br>☐ Other (please specify)<br>________________________.	☐ Car<br>☐ Public transport<br>☐ Walk/cycle<br>☐ Other<br>**OR**<br>☐ Home delivery	☐ Fresh fruit<br>☐ Fresh vegetables<br>☐ Grocery items (e.g. bread, milk, pasta, meat)<br>☐ Snack food (e.g. chips, chocolate)<br>☐ Soft drink<br>☐ Bottled water<br>☐ Hot fast food/takeaway (e.g. KFC, Chinese, pizza)<br>☐ Cold takeaway (e.g. sandwich, salad, sushi)<br>☐ Meal within sit down restaurant<br>☐ Other (please specify briefly) ____________________.	

FOOD PURCHASING DIARY

Day 12 continued

**Food purchase (FP) number/ receipt attached**<br><br>*(report for each different store visited)*	**Store name/address**<br><br>(minimum information: store name, street/location, suburb)	**Where were you prior to making this food purchase?**	**Primary mode of travel from the prior location to the food store.**	**What food was purchased**<br><br>Please keep and return receipt and note which day and food purchase number it relates to<br>**OR**<br>Fill in checklist below	**Amount spent $**
**# 3**<br><br>Receipt attached:<br>☐ Yes<br>☐ No	Store name:<br><br>Street number:<br><br>Street name/location:<br><br>Suburb:	☐ Home<br>☐ Work<br>☐ Other (please specify)<br>______________________.	☐ Car<br>☐ Public transport<br>☐ Walk/cycle<br>☐ Other<br>**OR**<br>☐ Home delivery	☐ Fresh fruit<br>☐ Fresh vegetables<br>☐ Grocery items (e.g. bread, milk, pasta)<br>☐ Snack food (e.g. chips, chocolate)<br>☐ Soft drink<br>☐ Bottled water<br>☐ Hot fast food/takeaway (e.g. KFC, Chinese, pizza)<br>☐ Cold takeaway (e.g. sandwich, salad, sushi)<br>☐ Meal within sit down restaurant<br>☐ Other (please specify briefly) __________________.	
**# 4**<br><br>Receipt attached:<br>☐ Yes<br>☐ No	Store name:<br><br>Street number:<br><br>Street name/location:<br><br>Suburb:	☐ Home<br>☐ Work<br>☐ Other (please specify)<br>______________________.	☐ Car<br>☐ Public transport<br>☐ Walk/cycle<br>☐ Other<br>**OR**<br>☐ Home delivery	☐ Fresh fruit<br>☐ Fresh vegetables<br>☐ Grocery items (e.g. bread, milk, pasta, meat)<br>☐ Snack food (e.g. chips, chocolate)<br>☐ Soft drink<br>☐ Bottled water<br>☐ Hot fast food/takeaway (e.g. KFC, Chinese, pizza)<br>☐ Cold takeaway (e.g. sandwich, salad, sushi)<br>☐ Meal within sit down restaurant<br>☐ Other (please specify briefly) __________________.	

FOOD PURCHASING DIARY

Day 13: ______________________ **No food purchase made on this day:** ☐

**Food purchase (FP) number/ receipt attached**<br><br>*(report for each different store visited)*	**Store name/address**<br><br>(<u>minimum information:</u> store name, street/location, suburb)	**Where were you prior to making this food purchase?**	**Primary mode of travel from the prior location to the food store.**	**What food was purchased**<br><br>Please keep and return receipt and note which day and food purchase number it relates to<br>**OR**<br>Fill in checklist below	**Amount spent $**
**# 1**<br><br>Receipt attached:<br>☐ Yes<br>☐ No	Store name:<br><br>Street number:<br><br>Street name/location:<br><br>Suburb:	☐ Home<br>☐ Work<br>☐ Other (please specify)<br>______________________.	☐ Car<br>☐ Public transport<br>☐ Walk/cycle<br>☐ Other<br>**OR**<br>☐ Home delivery	☐ Fresh fruit<br>☐ Fresh vegetables<br>☐ Grocery items (e.g. bread, milk, pasta)<br>☐ Snack food (e.g. chips, chocolate)<br>☐ Soft drink<br>☐ Bottled water<br>☐ Hot fast food/takeaway (e.g. KFC, Chinese, pizza)<br>☐ Cold takeaway (e.g. sandwich, salad, sushi)<br>☐ Meal within sit down restaurant<br>☐ Other (please specify briefly) ________________.	
**# 2**<br><br>Receipt attached:<br>☐ Yes<br>☐ No	Store name:<br><br>Street number:<br><br>Street name/location:<br><br>Suburb:	☐ Home<br>☐ Work<br>☐ Other (please specify)<br>______________________.	☐ Car<br>☐ Public transport<br>☐ Walk/cycle<br>☐ Other<br>**OR**<br>☐ Home delivery	☐ Fresh fruit<br>☐ Fresh vegetables<br>☐ Grocery items (e.g. bread, milk, pasta, meat)<br>☐ Snack food (e.g. chips, chocolate)<br>☐ Soft drink<br>☐ Bottled water<br>☐ Hot fast food/takeaway (e.g. KFC, Chinese, pizza)<br>☐ Cold takeaway (e.g. sandwich, salad, sushi)<br>☐ Meal within sit down restaurant<br>☐ Other (please specify briefly) ________________.	

FOOD PURCHASING DIARY

Day 13 continued

**Food purchase (FP) number/ receipt attached**<br><br>*(report for each different store visited)*	**Store name/address**<br><br>(minimum information: store name, street/location, suburb)	**Where were you prior to making this food purchase?**	**Primary mode of travel from the prior location to the food store.**	**What food was purchased**<br><br>Please keep and return receipt and note which day and food purchase number it relates to<br>**OR**<br>Fill in checklist below	**Amount spent $**
**# 3**<br><br>Receipt attached:<br>☐ Yes<br>☐ No	Store name:<br><br>Street number:<br><br>Street name/location:<br><br>Suburb:	☐ Home<br>☐ Work<br>☐ Other (please specify)<br>____________________.	☐ Car<br>☐ Public transport<br>☐ Walk/cycle<br>☐ Other<br>**OR**<br>☐ Home delivery	☐ Fresh fruit<br>☐ Fresh vegetables<br>☐ Grocery items (e.g. bread, milk, pasta)<br>☐ Snack food (e.g. chips, chocolate)<br>☐ Soft drink<br>☐ Bottled water<br>☐ Hot fast food/takeaway (e.g. KFC, Chinese, pizza)<br>☐ Cold takeaway (e.g. sandwich, salad, sushi)<br>☐ Meal within sit down restaurant<br>☐ Other (please specify briefly) ________________.	
**# 4**<br><br>Receipt attached:<br>☐ Yes<br>☐ No	Store name:<br><br>Street number:<br><br>Street name/location:<br><br>Suburb:	☐ Home<br>☐ Work<br>☐ Other (please specify)<br>____________________.	☐ Car<br>☐ Public transport<br>☐ Walk/cycle<br>☐ Other<br>**OR**<br>☐ Home delivery	☐ Fresh fruit<br>☐ Fresh vegetables<br>☐ Grocery items (e.g. bread, milk, pasta, meat)<br>☐ Snack food (e.g. chips, chocolate)<br>☐ Soft drink<br>☐ Bottled water<br>☐ Hot fast food/takeaway (e.g. KFC, Chinese, pizza)<br>☐ Cold takeaway (e.g. sandwich, salad, sushi)<br>☐ Meal within sit down restaurant<br>☐ Other (please specify briefly) ________________.	

FOOD PURCHASING DIARY

Day 14: ______________________ **(FINAL DAY)** **No food purchase made on this day:** ☐

**Food purchase (FP) number/ receipt attached**<br><br>*(report for each different store visited)*	**Store name/address**<br><br>(minimum information: store name, street/location, suburb)	**Where were you prior to making this food purchase?**	**Primary mode of travel from the prior location to the food store.**	**What food was purchased**<br><br>Please keep and return receipt and note which day and food purchase number it relates to<br>**OR**<br>Fill in checklist below	**Amount spent $**
**# 1**<br><br>Receipt attached:<br>☐ Yes<br>☐ No	Store name:<br><br>Street number:<br><br>Street name/location:<br><br>Suburb:	☐ Home<br>☐ Work<br>☐ Other (please specify)<br>________________________.	☐ Car<br>☐ Public transport<br>☐ Walk/cycle<br>☐ Other<br>**OR**<br>☐ Home delivery	☐ Fresh fruit<br>☐ Fresh vegetables<br>☐ Grocery items (e.g. bread, milk, pasta)<br>☐ Snack food (e.g. chips, chocolate)<br>☐ Soft drink<br>☐ Bottled water<br>☐ Hot fast food/takeaway (e.g. KFC, Chinese, pizza)<br>☐ Cold takeaway (e.g. sandwich, salad, sushi)<br>☐ Meal within sit down restaurant<br>☐ Other (please specify briefly) ____________________.	
**# 2**<br><br>Receipt attached:<br>☐ Yes<br>☐ No	Store name:<br><br>Street number:<br><br>Street name/location:<br><br>Suburb:	☐ Home<br>☐ Work<br>☐ Other (please specify)<br>________________________.	☐ Car<br>☐ Public transport<br>☐ Walk/cycle<br>☐ Other<br>**OR**<br>☐ Home delivery	☐ Fresh fruit<br>☐ Fresh vegetables<br>☐ Grocery items (e.g. bread, milk, pasta, meat)<br>☐ Snack food (e.g. chips, chocolate)<br>☐ Soft drink<br>☐ Bottled water<br>☐ Hot fast food/takeaway (e.g. KFC, Chinese, pizza)<br>☐ Cold takeaway (e.g. sandwich, salad, sushi)<br>☐ Meal within sit down restaurant<br>☐ Other (please specify briefly) ____________________.	

FOOD PURCHASING DIARY

Day 14 continued (FINAL DAY)

**Food purchase (FP) number/ receipt attached**<br><br>*(report for each different store visited)*	**Store name/address**<br><br>(<u>minimum information:</u> store name, street/location, suburb)	**Where were you prior to making this food purchase?**	**Primary mode of travel from the prior location to the food store.**	**What food was purchased**<br><br>Please keep and return receipt and note which day and food purchase number it relates to<br>**OR**<br>Fill in checklist below	**Amount spent $**
**# 3**<br><br>Receipt attached:<br>☐ Yes<br>☐ No	Store name:<br><br>Street number:<br><br>Street name/location:<br><br>Suburb:	☐ Home<br>☐ Work<br>☐ Other (please specify)<br>____________________.	☐ Car<br>☐ Public transport<br>☐ Walk/cycle<br>☐ Other<br>**OR**<br>☐ Home delivery	☐ Fresh fruit<br>☐ Fresh vegetables<br>☐ Grocery items (e.g. bread, milk, pasta)<br>☐ Snack food (e.g. chips, chocolate)<br>☐ Soft drink<br>☐ Bottled water<br>☐ Hot fast food/takeaway (e.g. KFC, Chinese, pizza)<br>☐ Cold takeaway (e.g. sandwich, salad, sushi)<br>☐ Meal within sit down restaurant<br>☐ Other (please specify briefly) ________________.	
**# 4**<br><br>Receipt attached:<br>☐ Yes<br>☐ No	Store name:<br><br>Street number:<br><br>Street name/location:<br><br>Suburb:	☐ Home<br>☐ Work<br>☐ Other (please specify)<br>____________________.	☐ Car<br>☐ Public transport<br>☐ Walk/cycle<br>☐ Other<br>**OR**<br>☐ Home delivery	☐ Fresh fruit<br>☐ Fresh vegetables<br>☐ Grocery items (e.g. bread, milk, pasta, meat)<br>☐ Snack food (e.g. chips, chocolate)<br>☐ Soft drink<br>☐ Bottled water<br>☐ Hot fast food/takeaway (e.g. KFC, Chinese, pizza)<br>☐ Cold takeaway (e.g. sandwich, salad, sushi)<br>☐ Meal within sit down restaurant<br>☐ Other (please specify briefly) ________________.	

ADDITIONAL PAGES FOLLOW

- Please use the following pages to record purchases on days where you visited more than four separate stores to make food purchases.
- Indicate in the first column which day number the food purchase corresponds to.
- If you did not make more than food separate food purchases on any one day, you do not need to complete these pages. Please skip ahead to the short questionnaire on yourself.

FOOD PURCHASING DIARY

**Day number of additional food purchase / receipt attached**<br><br>*(report for each different store visited)*	**Store name/address**<br><br>(<u>minimum information:</u> store name, street/location, suburb)	**Where were you prior to making this food purchase?**	**Primary mode of travel from the prior location to the food store.**	**What food was purchased**<br><br>Please keep and return receipt and note which day and food purchase number it relates to<br>**OR**<br>Fill in checklist below	**Amount spent $**
**Day:**<br><br>Receipt attached:<br>☐ Yes<br>☐ No	Store name:<br><br>Street number:<br><br>Street name/location:<br><br>Suburb:	☐ Home<br>☐ Work<br>☐ Other (please specify)<br>________________________.	☐ Car<br>☐ Public transport<br>☐ Walk/cycle<br>☐ Other<br>**OR**<br>☐ Home delivery	☐ Fresh fruit<br>☐ Fresh vegetables<br>☐ Grocery items (e.g. bread, milk, pasta)<br>☐ Snack food (e.g. chips, chocolate)<br>☐ Soft drink<br>☐ Bottled water<br>☐ Hot fast food/takeaway (e.g. KFC, Chinese, pizza)<br>☐ Cold takeaway (e.g. sandwich, salad, sushi)<br>☐ Meal within sit down restaurant<br>☐ Other (please specify briefly) ____________________.	
**Day:**<br><br>Receipt attached:<br>☐ Yes<br>☐ No	Store name:<br><br>Street number:<br><br>Street name/location:<br><br>Suburb:	☐ Home<br>☐ Work<br>☐ Other (please specify)<br>________________________.	☐ Car<br>☐ Public transport<br>☐ Walk/cycle<br>☐ Other<br>**OR**<br>☐ Home delivery	☐ Fresh fruit<br>☐ Fresh vegetables<br>☐ Grocery items (e.g. bread, milk, pasta, meat)<br>☐ Snack food (e.g. chips, chocolate)<br>☐ Soft drink<br>☐ Bottled water<br>☐ Hot fast food/takeaway (e.g. KFC, Chinese, pizza)<br>☐ Cold takeaway (e.g. sandwich, salad, sushi)<br>☐ Meal within sit down restaurant<br>☐ Other (please specify briefly) ____________________.	

FOOD PURCHASING DIARY

**Day number of additional food purchase / receipt attached**<br><br>*(report for each different store visited)*	**Store name/address**<br><br>(<u>minimum information:</u> store name, street/location, suburb)	**Where were you prior to making this food purchase?**	**Primary mode of travel from the prior location to the food store.**	**What food was purchased**<br><br>Please keep and return receipt and note which day and food purchase number it relates to<br>**OR**<br>Fill in checklist below	**Amount spent $**
**Day:**<br><br>Receipt attached:<br>☐ Yes<br>☐ No	Store name:<br><br>Street number:<br><br>Street name/location:<br><br>Suburb:	☐ Home<br>☐ Work<br>☐ Other (please specify)<br>______________________.	☐ Car<br>☐ Public transport<br>☐ Walk/cycle<br>☐ Other<br>**OR**<br>☐ Home delivery	☐ Fresh fruit<br>☐ Fresh vegetables<br>☐ Grocery items (e.g. bread, milk, pasta)<br>☐ Snack food (e.g. chips, chocolate)<br>☐ Soft drink<br>☐ Bottled water<br>☐ Hot fast food/takeaway (e.g. KFC, Chinese, pizza)<br>☐ Cold takeaway (e.g. sandwich, salad, sushi)<br>☐ Meal within sit down restaurant<br>☐ Other (please specify briefly) ________________.	
**Day:**<br><br>Receipt attached:<br>☐ Yes<br>☐ No	Store name:<br><br>Street number:<br><br>Street name/location:<br><br>Suburb:	☐ Home<br>☐ Work<br>☐ Other (please specify)<br>______________________.	☐ Car<br>☐ Public transport<br>☐ Walk/cycle<br>☐ Other<br>**OR**<br>☐ Home delivery	☐ Fresh fruit<br>☐ Fresh vegetables<br>☐ Grocery items (e.g. bread, milk, pasta, meat)<br>☐ Snack food (e.g. chips, chocolate)<br>☐ Soft drink<br>☐ Bottled water<br>☐ Hot fast food/takeaway (e.g. KFC, Chinese, pizza)<br>☐ Cold takeaway (e.g. sandwich, salad, sushi)<br>☐ Meal within sit down restaurant<br>☐ Other (please specify briefly) ________________.	

FOOD PURCHASING DIARY

**Day number of additional food purchase / receipt attached**<br><br>*(report for each different store visited)*	**Store name/address**<br><br>(minimum information: store name, street/location, suburb)	**Where were you prior to making this food purchase?**	**Primary mode of travel from the prior location to the food store.**	**What food was purchased**<br><br>Please keep and return receipt and note which day and food purchase number it relates to<br>**OR**<br>Fill in checklist below	**Amount spent $**
**Day:**<br><br>Receipt attached:<br>☐ Yes<br>☐ No	Store name:<br><br>Street number:<br><br>Street name/location:<br><br>Suburb:	☐ Home<br>☐ Work<br>☐ Other (please specify)<br>______________________.	☐ Car<br>☐ Public transport<br>☐ Walk/cycle<br>☐ Other<br>**OR**<br>☐ Home delivery	☐ Fresh fruit<br>☐ Fresh vegetables<br>☐ Grocery items (e.g. bread, milk, pasta)<br>☐ Snack food (e.g. chips, chocolate)<br>☐ Soft drink<br>☐ Bottled water<br>☐ Hot fast food/takeaway (e.g. KFC, Chinese, pizza)<br>☐ Cold takeaway (e.g. sandwich, salad, sushi)<br>☐ Meal within sit down restaurant<br>☐ Other (please specify briefly) _________________.	
**Day:**<br><br>Receipt attached:<br>☐ Yes<br>☐ No	Store name:<br><br>Street number:<br><br>Street name/location:<br><br>Suburb:	☐ Home<br>☐ Work<br>☐ Other (please specify)<br>______________________.	☐ Car<br>☐ Public transport<br>☐ Walk/cycle<br>☐ Other<br>**OR**<br>☐ Home delivery	☐ Fresh fruit<br>☐ Fresh vegetables<br>☐ Grocery items (e.g. bread, milk, pasta, meat)<br>☐ Snack food (e.g. chips, chocolate)<br>☐ Soft drink<br>☐ Bottled water<br>☐ Hot fast food/takeaway (e.g. KFC, Chinese, pizza)<br>☐ Cold takeaway (e.g. sandwich, salad, sushi)<br>☐ Meal within sit down restaurant<br>☐ Other (please specify briefly) _________________.	

FOOD PURCHASING DIARY

**Day number of additional food purchase / receipt attached**<br><br>*(report for each different store visited)*	**Store name/address**<br><br>(<u>minimum information:</u> store name, street/location, suburb)	**Where were you prior to making this food purchase?**	**Primary mode of travel from the prior location to the food store.**	**What food was purchased**<br><br>Please keep and return receipt and note which day and food purchase number it relates to<br>**OR**<br>Fill in checklist below	**Amount spent $**
**Day:**<br><br>Receipt attached:<br>☐ Yes<br>☐ No	Store name:<br><br>Street number:<br><br>Street name/location:<br><br>Suburb:	☐ Home<br>☐ Work<br>☐ Other (please specify)<br>________________.	☐ Car<br>☐ Public transport<br>☐ Walk/cycle<br>☐ Other<br>**OR**<br>☐ Home delivery	☐ Fresh fruit<br>☐ Fresh vegetables<br>☐ Grocery items (e.g. bread, milk, pasta)<br>☐ Snack food (e.g. chips, chocolate)<br>☐ Soft drink<br>☐ Bottled water<br>☐ Hot fast food/takeaway (e.g. KFC, Chinese, pizza)<br>☐ Cold takeaway (e.g. sandwich, salad, sushi)<br>☐ Meal within sit down restaurant<br>☐ Other (please specify briefly) ____________.	
**Day:**<br><br>Receipt attached:<br>☐ Yes<br>☐ No	Store name:<br><br>Street number:<br><br>Street name/location:<br><br>Suburb:	☐ Home<br>☐ Work<br>☐ Other (please specify)<br>________________.	☐ Car<br>☐ Public transport<br>☐ Walk/cycle<br>☐ Other<br>**OR**<br>☐ Home delivery	☐ Fresh fruit<br>☐ Fresh vegetables<br>☐ Grocery items (e.g. bread, milk, pasta, meat)<br>☐ Snack food (e.g. chips, chocolate)<br>☐ Soft drink<br>☐ Bottled water<br>☐ Hot fast food/takeaway (e.g. KFC, Chinese, pizza)<br>☐ Cold takeaway (e.g. sandwich, salad, sushi)<br>☐ Meal within sit down restaurant<br>☐ Other (please specify briefly) ____________.	

32927

Individual Questionnaire

1. Are you?

☐ Male

☐ Female

2. How old are you?

☐ 18-24 years

☐ 25-34 years

☐ 35-44 years

☐ 45-54 years

☐ 55-64 years

☐ 65+ years

3. Where were you born?

☐ Australia

☐ Overseas

4. With which of the following groups do you identify yourself culturally?

☐ European/Caucasian

☐ East/South East Asian (Thai, Chinese)

☐ African

☐ South Asia (India, Sri Lanka)

☐ Other, please specify ______________________________

5. Which of the following best describes your current relationship status?

☐ Married/de facto

☐ Separated/divorced

☐ Widowed

☐ Never married

6. How many people of the following age groups (including yourself) live in your household? Shade the corresponding box for each age group.

	Number of people in the household						
	Zero	1	2	3	4	5	6 or more
0-6 years	○	○	○	○	○	○	○
6-12 years	○	○	○	○	○	○	○
12-18 years	○	○	○	○	○	○	○
18-25 years	○	○	○	○	○	○	○
Over 25 years	○	○	○	○	○	○	○

32927

7. What is the HIGHEST qualification you and your partner/spouse, if applicable, have completed?

Self:

- ☐ Did not complete high school
- ☐ Year 12 or equivalent (e.g. Higher School Certificate)
- ☐ Trade/apprenticeship/certificate or diploma (e.g. hairdresser, chef)
- ☐ University or higher degree

Partner:

- ☐ Did not complete high school
- ☐ Year 12 or equivalent (e.g. Higher School Certificate)
- ☐ Trade/apprenticeship/certificate or diploma (e.g. hairdresser, chef)
- ☐ University or higher degree
- ☐ No partner/spouse

8. If you are employed, please record:

Your main occupation:

____

Number of hours per week you spend in paid employment:

☐☐

The post code of your current work location:

☐☐☐☐

9. What is your estimated total household income before tax? This is the combined total income of all persons living in your household.

- ☐ <= $25,999 per year (<=$500 per week)
- ☐ $26,000 - $38,999 per year ($500 - $700 per week)
- ☐ $39,000 - $51,999 per year ($750 - $1000 per week)
- ☐ $52,000 - $64,999 per year ($1000-$1250 per week)
- ☐ $65,000 - $77,999 per year ($1250 - $1500 per week)
- ☐ $78,000 - $90,999 per year ($1500 - $1750 per week)
- ☐ $91,000 - $103,999 per year ($1750 - $2000 per week)
- ☐ $104,000 - $116,999 per year ($2000 - $2250 per week)
- ☐ $117,000 - $129,999 per year ($2250 - $2500 per week)
- ☐ $130,000 - $142,999 per year ($2500 - $2750 per week)
- ☐ $142,000 - $155,999 per year ($2750 - $3000 per week)
- ☐ >= $156,000 per year (>$3000 per week)

32927

10. On a scale of 1-7 how would you rate your overall health?(Please circle)

1 2 3 4 5 6 7

Very poor ... Excellent

11. What is your height and weight?

Height (cm) [ ][ ][ ]

Weight (kg) [ ][ ][ ]

12. Does your health limit your ability to lift or carry groceries?

☐ Yes

☐ No

13. Do you always have access to a car to do your food shopping?

☐ Yes

☐ No

14. How much do you agree or disagree with the following statements? (Please circle)

a. Fruits and vegetables in my neighbourhood are affordable:

1 2 3 4 5 6 7

Strongly disagree ... Strongly agree

b. Shops that sell fruits and vegetables are easily accessible in my neighbourhood:

1 2 3 4 5 6 7

Strongly disagree ... Strongly agree

c. Fast food/takeaway foods in my neighbourhood are affordable:

1 2 3 4 5 6 7

Strongly disagree ... Strongly agree

d. Shops that sell fast food/takeaway foods are easily accessible (i.e. delivery options, variety of shops close to home) in my neighbourhood:

1 2 3 4 5 6 7

Strongly disagree ... Strongly agree

32927

15. How important are the following factors in influencing where you shop for food? (Please circle)

a. Food store is easily accessible from my home:

1 2 3 4 5 6 7
Not at all — Very important

b. Food store is easily accessible from my work:

1 2 3 4 5 6 7
Not at all — Very important

c. Food store is close to other shops and services (e.g. post office) that I use:

1 2 3 4 5 6 7
Not at all — Very important

d. Food store is close to child's school, social activities or other frequently visited locations (e.g. gym):

1 2 3 4 5 6 7
Not at all — Very important

e. Product range within food store:

1 2 3 4 5 6 7
Not at all — Very important

f. Parking near food store:

1 2 3 4 5 6 7
Not at all — Very important

g. Good public transport to/from the food store:

1 2 3 4 5 6 7
Not at all — Very important

h. Rewards programs:

1 2 3 4 5 6 7
Not at all — Very important

i. Opening hours of store:

1 2 3 4 5 6 7
Not at all — Very important

32927

16. How much do you agree or disagree with the following statements?
(Please circle)

a. I prefer to shop in a store that is part of a larger chain (e.g. Coles, McDonalds):

	1	2	3	4	5	6	7
Strongly disagree							Strongly agree

b. I prefer to shop in a store that is independently owned (e.g. local greengrocer):

	1	2	3	4	5	6	7
Strongly disagree							Strongly agree

c. I prefer to buy food that can be purchased quickly:

	1	2	3	4	5	6	7
Strongly disagree							Strongly agree

d. I prefer to buy food that requires little to no preparation time:

	1	2	3	4	5	6	7
Strongly disagree							Strongly agree

e. I avoid food stores that are in areas I do not feel safe visiting:

	1	2	3	4	5	6	7
Strongly disagree							Strongly agree

f. I make the majority of my food purchasing decisions once I am within the store:

	1	2	3	4	5	6	7
Strongly disagree							Strongly agree

32927

17. How important are the following factors in influencing what foods you buy? (Please circle)

a. Price of food:

	1	2	3	4	5	6	7	
Not at all								Very important

b. Freshness/quality of food:

	1	2	3	4	5	6	7	
Not at all								Very important

c. Taste preference:

	1	2	3	4	5	6	7	
Not at all								Very important

d. Healthiness of food:

	1	2	3	4	5	6	7	
Not at all								Very important

e. Special dietary requirements:

	1	2	3	4	5	6	7	
Not at all								Very important

f. Food preferences of other family/household members:

	1	2	3	4	5	6	7	
Not at all								Very important

32927

18. How much do you agree or disagree with the following statements:
(Please circle)

a. Fruits are a convenient snack:

	1	2	3	4	5	6	7	
Strongly disagree								Strongly agree

b. Fruits and vegetables taste good:

	1	2	3	4	5	6	7	
Strongly disagree								Strongly agree

c. My friends like to eat a lot of fruits and vegetables:

	1	2	3	4	5	6	7	
Strongly disagree								Strongly agree

d. My family likes to eat a lot of fruits and vegetables:

	1	2	3	4	5	6	7	
Strongly disagree								Strongly agree

e. Fast food/takeaway food is a convenient snack:

	1	2	3	4	5	6	7	
Strongly disagree								Strongly agree

f. Fast food/takeaway food tastes good:

	1	2	3	4	5	6	7	
Strongly disagree								Strongly agree

g. My friends like to eat a lot of fast food/takeaway food:

	1	2	3	4	5	6	7	
Strongly disagree								Strongly agree

h. My family likes to eat a lot of fast food/takeaway food:

	1	2	3	4	5	6	7	
Strongly disagree								Strongly agree

32927

19. What is the nearest large supermarket to where you live? (e.g. Coles, Woolworths, ALDI, IGA). Please provide the name of the supermarket, the street it is on and the suburb it is in.

Shop name:

____

Street:

____

Suburb:

____

20. Do you regularly purchase food at this supermarket?

☐ Yes

☐ No

21. If you answered Yes to question 20, please list three reasons why you choose to shop in your store.

____

____

____

If you answered No to question 20, please list three reasons why you do not shop at this store.

____

____

____

Thankyou again for participating in this study.

Please mail this document back in the reply paid envelope supplied.

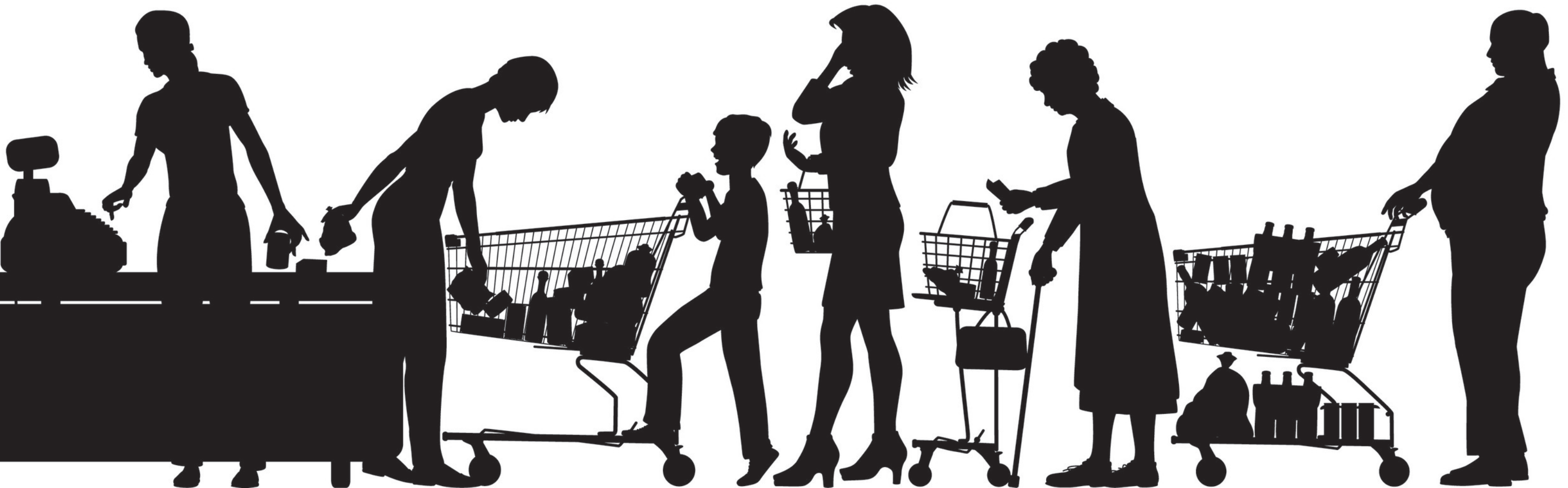

Supplement: Supplementary file 1 — Additional file 1. Data collection tool. [file 12942_2017_82_MOESM1_ESM.pdf]
