# Supplementary material for: Where do people purchase food? A novel approach to investigating food purchasing locations
Source: Int J Health Geogr. 2017 Mar 7;16:9. doi: 10.1186/s12942-017-0082-z (PMC5341394; doi:10.1186/s12942-017-0082-z)
Supplement: Supplementary file 2 — Additional file 2. Supplementary tables. [file 12942_2017_82_MOESM2_ESM.docx]

## Table S1. Food items and their definition

| **Item** | **Definition** |
| --- | --- |
| Fresh fruit | All fresh fruit items that are either as individual items (e.g. a single apple) or as bulk (e.g. 6 pack of Kiwi fruit). Do not included canned or dried fruit in this category. |
| Fresh vegetables | All fresh vegetable items that are either as individual items (e.g. a lettuce) or as bulk (e.g. 1kg of carrots). Do not included canned or dried vegetables in this category. |
| Grocery items | This includes household grocery items such as bread, milk, cheese, pasta, rice, tinned fish, butter, canned fruit, meat, breakfast cereal, yoghurt etc. |
| Snack food | Potato/corn chips, chocolate, confectionery, muesli bars, popcorn, pretzels, pastries (e.g. cinnamon bun), ice cream etc. |
| Bottled water | This includes all bottled natural water (still, free of flavours  Etc.). |
| Soft drink | This includes all soft drinks (both sugar sweetened and diet varieties), sports drinks, energy drinks etc. |
| Hot fast food/takeaway | Examples include hot chips, pizza, meat pie, hamburger, souvlaki, fish & chips, fried/roast chicken, fried rice, curry, etc. |
| Cold takeaway | Examples include sandwiches, salads, sushi etc. |
| Meal within sit down restaurant | This includes all eat in occasions in a full-table service sit down restaurant. It does not include occasions such as eating within a McDonald’s restaurant (which should be recorded under hot fast food/takeaway). |
| Other | Please specify any other item that is not in the listed above here. Additionally, if you are unsure how to classify an item, please list it here. |

## Table S2. Post-estimation pairwise comparison for distance from home to food purchase location by neighbourhood type (Model 2)

|  | **All purchases** | | **Purchases made when home was the origin** | | **Purchases made at supermarkets** | |
| --- | --- | --- | --- | --- | --- | --- |
| **Neighbourhood** | **Coef. (95% CI)** | **p. value** | **Coef. (95% CI)** | **p. value** | **Coef. (95% CI)** | **p. value** |
| Low SES-High access vs Low SES-Low access (REF) | -0.25 (-0.94, 0.44) | 0.477 | -0.26 (-0.85, 0.32) | 0.376 | -0.46 (-1.33, 0.40) | 0.293 |
| High SES-Low access vs Low SES-Low access (REF) | 0.70 (0.04, 1.36) | 0.037 | 0.57 (0.07, 1.08) | 0.025 | 0.75 (-0.08, 1.58) | 0.077 |
| High SES-High access vs Low SES-Low access (REF) | -0.09 (-0.76, 0.59) | 0.800 | -0.19 (-0.72, 0.34) | 0.477 | -0.13 (-0.96, 0.71) | 0.767 |
| High SES-Low access vs Low SES-High access (REF) | 0.95 (0.25, 1.65) | 0.008 | 0.84 (0.26, 1.41) | 0.004 | 1.21 (0.35, 2.07) | 0.006 |
| High SES-High access vs Low SES-High access (REF) | 0.16 (-0.55, 0.88) | 0.654 | 0.07 (-0.52, 0.66) | 0.812 | 0.34 (-0.53, 1.20) | 0.446 |
| High SES-High access vs High SES-Low access (REF) | -0.79 (-1.44, -0.13) | 0.018 | -0.77 (-1.26, -0.27) | 0.002 | -0.87 (-1.70, -0.05) | 0.038 |
